# Estimating quantitative genetic parameters in wild populations: a comparison of pedigree and genomic approaches

**DOI:** 10.1111/mec.12827

**Published:** 2014-06-26

**Authors:** Camillo Bérénos, Philip A Ellis, Jill G Pilkington, Josephine M Pemberton

**Affiliations:** Institute of Evolutionary Biology, School of Biological Sciences, University of EdinburghWest Mains Road, Edinburgh, EH9 3JT, UK

**Keywords:** body size, genetic architecture, genomic relatedness, heritability, maternal genetic effect, SNP genotyping

## Abstract

The estimation of quantitative genetic parameters in wild populations is generally limited by the accuracy and completeness of the available pedigree information. Using relatedness at genomewide markers can potentially remove this limitation and lead to less biased and more precise estimates. We estimated heritability, maternal genetic effects and genetic correlations for body size traits in an unmanaged long-term study population of Soay sheep on St Kilda using three increasingly complete and accurate estimates of relatedness: (i) Pedigree 1, using observation-derived maternal links and microsatellite-derived paternal links; (ii) Pedigree 2, using SNP-derived assignment of both maternity and paternity; and (iii) whole-genome relatedness at 37 037 autosomal SNPs. In initial analyses, heritability estimates were strikingly similar for all three methods, while standard errors were systematically lower in analyses based on Pedigree 2 and genomic relatedness. Genetic correlations were generally strong, differed little between the three estimates of relatedness and the standard errors declined only very slightly with improved relatedness information. When partitioning maternal effects into separate genetic and environmental components, maternal genetic effects found in juvenile traits increased substantially across the three relatedness estimates. Heritability declined compared to parallel models where only a maternal environment effect was fitted, suggesting that maternal genetic effects are confounded with direct genetic effects and that more accurate estimates of relatedness were better able to separate maternal genetic effects from direct genetic effects. We found that the heritability captured by SNP markers asymptoted at about half the SNPs available, suggesting that denser marker panels are not necessarily required for precise and unbiased heritability estimates. Finally, we present guidelines for the use of genomic relatedness in future quantitative genetics studies in natural populations.

## Introduction

Knowledge of the genetic architecture underpinning phenotypic variation in natural populations is pivotal for our understanding of evolutionary processes. Classical quantitative genetics assumes the infinitesimal model, under which trait variation is controlled by alleles of small effect at many loci as well as environmental variation, and to date, extensive searches for quantitative trait loci in various taxa give strong support for this model (Mackay & Lyman 2005; Yang *et al*. 2010). Consequently, estimating additive genetic variance for individual traits and other sources of variance such as indirect genetic effects, especially maternal effects, is likely to be the most effective approach in estimating the potential for a population to evolve in response to selection (Bijma & Wade 2008; Kruuk *et al*. 2008).

Classical quantitative genetics uses information on the relatedness between individuals, often derived from a pedigree, to estimate what proportion of trait variance is explained by genes (heritability) and to what extent there is covariance between pairs of traits (genetic correlations). In recent years, there has been a marked increase in the application of this approach in wild populations (Kruuk *et al*. 2008), due to the accumulation of large data sets of phenotypes in several long-term studies, the acquisition of pedigrees (using observation or molecular parentage inference) and the adoption of the animal breeders' mixed model framework, the ‘animal model’ (Henderson 1984) which allows researchers to use all available phenotypic data from complex, unbalanced pedigrees.

To date, the precision with which quantitative genetic parameters, such as heritability and genetic correlations, can be estimated in the wild has been constrained by the accuracy and completeness of the available pedigree information. Accuracy can be compromised when pedigree links are inferred using observational data (e.g. due to misidentified mother–young associations in the field, undiscovered extra-pair paternity in passerines (Griffith *et al*. 2002)) but also when marker-based parentage estimation is used to infer parentage, as the discriminatory power of commonly used markers such as microsatellites may not be sufficient (Sardell *et al*. 2010), thus introducing error (Walling *et al*. 2010). In addition, wild pedigrees often suffer from substantial rates of missing parentage data as a result of immigration and incomplete sampling of candidate parents (Pemberton 2008).

Intuitively, it may be expected that pedigree errors lead to a downward bias of both heritability estimates and genetic covariances. However, their effects are poorly understood, especially in the context of wild populations. In dairy cattle, estimates of maternal and direct genetic effects increased with decreasing simulated pedigree errors (Senneke *et al*. 2004), while estimated breeding values were biased in the presence of paternity errors (Israel & Weller 2000). In wild blue tit (*Cyanistes caeruleus*) populations, identification of extra-pair paternities led to higher heritability estimates of tarsus length and body mass, but pedigrees with 20% paternity errors only led to heritability underestimates of 5% (Charmantier & Reale 2005). More surprisingly, social pedigrees sometimes retrieve higher heritability estimates than the correct genetic pedigrees, which is explained by sampling error due to low sample sizes, commonplace in wild populations (Charmantier & Reale 2005). In our Soay sheep (*Ovis aries*) population, simulations showed that, in the absence of maternal effects, downward bias of heritability increased with increasing rate of pedigree errors (Morrissey *et al*. 2007). Even less is known about how pedigree errors affect the estimation of genetic covariances and correlations, although the previously mentioned simulation study showed that estimates of genetic correlations were not influenced much by pedigree errors in the Soay sheep (Morrissey *et al*. 2007).

While maternal effects are often considered to be a form of common environment effect, a mother's genotype can also contribute to offspring phenotypic variation. Such maternal genetic effects have been widely documented in livestock (Dodenhoff *et al*. 1998), but data in natural populations are relatively scarce (Wilson *et al*. 2005; Kruuk & Hadfield 2007; Rasanen & Kruuk 2007). In some systems, such as passerine birds, where most data come from full-sib and parent–offspring relationships, the natural pedigree structure limits the power to detect maternal (genetic) effects without multigenerational cross-fostering experiments. In ungulate species with polygynous mating systems such as the Soay sheep, pedigree structures are better suited to detect maternal genetic effects; however, inaccurate pedigrees could adversely affect power to detect maternal genetic effects and thus partially explain why they are so rarely found (Morrissey *et al*. 2007). When simulating a complex genetic architecture in Soay sheep (Morrissey *et al*. 2007), estimates of maternal genetic effects were not influenced by paternity errors, but a substantial upwards bias in heritability estimates was observed in models using the pedigree with the fewest false assignments. This could be explained by the fact that in these simulations, as in most pedigrees, two types of errors are traded off with each other. The pedigree with the lowest rate of paternity errors also displayed the highest rate of missing links (Morrissey *et al*. 2007). Due to the difficulty of identifying fathers, many wild pedigrees have an imbalance in parental links, with many more mothers than fathers identified, and so may be especially prone to biases in the estimation of maternal genetic effects.

While pedigree-based methods have enabled extensive research in the quantitative genetics of wild populations, recent advances in high-density genotyping offer the possibility of yet more precision. Superior genotyping information enables substantial improvements to the completeness and accuracy of pedigrees, reducing or eliminating many of the problems outlined in the previous paragraphs. Furthermore, even when pedigrees are perfect, actual relationships between individuals in a populations vary around the pedigree-predicted value due to segregation and recombination (Hill & Weir 2011), and if detectable, this variation can be informative for estimating trait covariance even with individuals of the same pedigree relatedness (Visscher *et al*. 2006).

When estimating quantitative genetic parameters using marker data, the genomic relatedness (also known as realized relatedness) would ideally be estimated at all causal loci underlying trait variance. As causal loci are unlikely to be among the genotyped markers, the accuracy of heritability estimates is dependent on linkage disequilibrium (LD) between causal loci and the genotyped markers at which relatedness is estimated (Yang *et al*. 2010). Estimates of heritability using genomic relatedness at hundreds of thousands of whole-genome markers in nominally unrelated individuals, for example, humans, are typically substantially lower than known heritabilities (Yang *et al*. 2010, 2011b), which is most parsimoniously explained by imperfect LD between causal and marker loci. Contrasting results are found in dairy cattle, where relatedness at common SNPs on commercially available 50K SNP chips explains much of the pedigree heritability for production traits (Jensen *et al*. 2012; Haile-Mariam *et al*. 2013). The difference in the proportion of genetic variance captured by SNPs in the human and cattle studies can be explained by two factors: first, the effective population size is much larger in humans than in cattle, causing LD to be much higher in cattle than humans, and second, the variance in relatedness of the genotyped samples, which is higher in cattle samples [which are typically genotyped for genomic prediction (Meuwissen *et al*. 2001)] than in humans [which are typically genotyped for genomewide association studies (GWAS) (Yang *et al*. 2010)]. While the potential applications are of great interest to evolutionary biologists, the relationship between genomic and pedigree-based heritability has not been thoroughly investigated in many other species to date (Lee *et al*. 2010; Gay *et al*. 2013; Stanton-Geddes *et al*. 2013), and studies examining whether genetic correlations can be successfully estimated in natural populations using dense marker panels are lacking (Lee *et al*. 2012; Vattikuti *et al*. 2012).

Estimating quantitative genetic parameters in ecological data sets using genomewide markers is still in its infancy (Robinson *et al*. 2013; Santure *et al*. 2013). Potential benefits could be more substantial in natural populations than has been previously shown in livestock (Jensen *et al*. 2012; Haile-Mariam *et al*. 2013) and human genetics (Visscher *et al*. 2006; Yang *et al*. 2010; Zaitlen *et al*. 2013). Environmental heterogeneity, small sample sizes and confounding between environmental and genetic effects characterize populations under natural conditions, potentially masking or inflating genetic effects if relatedness cannot be estimated accurately due to missing or erroneous links in the pedigree (Kruuk & Hadfield 2007).

In this study, we estimate the genetic and environmental variance components for five body size traits in a population of feral Soay sheep on Hirta, St. Kilda which has been subject to intensive individual-based study since 1985. We compare heritability, genetic correlations, maternal genetic and maternal environmental effect estimates obtained using (i) a pre-existing pedigree with paternities identified using a panel of microsatellites; (ii) a new pedigree based on a more powerful panel of SNP markers; and (iii) genomic relatedness derived from a 50K SNP chip. Body size traits are good candidates for exploring the effects of pedigree improvements and the performance of marker-based heritability in this study system. Body size is known to be heritable (Milner *et al*. 2000; Wilson *et al*. 2007), heritability increases with age (Wilson *et al*. 2007), and body size is influenced by maternal (genetic) effects in juveniles (Wilson *et al*. 2005, 2007) and is positively associated with survival and reproductive success (Coltman *et al*. 1999; Milner *et al*. 1999). Although there is some evidence for major effect QTL in Soay sheep (Beraldi *et al*. 2007), body size is likely to be highly polygenic, influenced by many loci with alleles of small effect, as it is in many other mammal species (Goddard & Hayes 2009; Yang *et al*. 2010).

The objectives of the study were therefore to determine: (i) the effect of a major improvement in pedigree completeness and accuracy on estimates of heritability and genetic correlations; (ii) if improved estimates of relatedness lead to better separation of maternal genetic and nongenetic maternal effects from direct genetic effects; (iii) the effect of improved estimation of relatedness on uncertainty of maternal effects and heritability estimates; and (iv) how much of the genetic variance as estimated by the best available pedigree is captured by relatedness at a dense panel of SNP loci. With these objectives, we aim to consider prospects for genomic-relatedness-based quantitative genetic studies of natural populations in the future.

## Methods

### Study population, phenotypic data and sampling

The Soay sheep is a primitive breed that has been living on the island of Soay, in the St. Kilda archipelago, NW Scotland, for thousands of years in a largely unmanaged state. The population on Hirta has been unmanaged since its introduction from Soay in 1932 (Clutton-Brock *et al*. 2004). Sheep resident in the Village Bay area of Hirta, comprising one-third of the Hirta population, have been the subject of intensive study since 1985 (Clutton-Brock *et al*. 2004).

The majority of lambs are ear-tagged and weighed within a couple of days of birth. Each August, approximately two-thirds of the resident population is trapped. At capture, body size traits which are measured include foreleg, hindleg and body weight. Winter mortality is monitored, and the left foreleg and both jawbones are collected, cleaned and stored. Body size traits measured from this *post-mortem* skeletal material include metacarpal length (mm) and jaw length (mm). More details about how these traits are measured can be found in Beraldi *et al*. 2007.

At first live capture, all sheep are ear-punched before ear tagging, and all sheep captured live are blood sampled into lithium heparin tubes, with the blood separated into plasma and buffy coat prior to freezing at −20 °C. A sample of ear tissue is also taken from all sheep when found dead, generally providing high-quality DNA for genotyping; in some cases, DNA is also available from muscle samples collected early in the study.

### Genotyping, pedigree construction and estimation of genomic relatedness

We attempted to extract suitable DNA (20 μL at 50ng/μL) from all individuals alive in the study population since 1990. DNA was extracted from ear punches and *post-mortem* ear samples using the Qiagen DNeasy 96 Blood and Tissue kit using the recommended protocol, except that final elution was in 2 × 50 μL elution buffer. For individuals, where no ear material was available, we extracted DNA from buffy coat samples using the Qiagen DNeasy Blood and Tissue kit, using the same final elution volumes; in a few individuals for which only *post-mortem* muscle was available, we extracted DNA using a standard phenol–chloroform method. DNA concentration was quantified using pico green (dsDNA BR Assay Kit, Invitrogen), and samples in the range 20–40 ng/μL were vacuum concentrated to achieve the desired final concentration. Samples below 20 ng/μL were not used further.

Genotyping was performed using the Ovine SNP50 BeadChip (Illumina) using an iScan instrument at the Wellcome Trust Clinical Research Facility Genetics Core (Edinburgh, UK). A total of 54 241 single-nucleotide polymorphisms (SNPs) distributed throughout the genome were genotyped. Results were inspected in genomestudio (Illumina). Most loci were clustered automatically, but 634 SNPs for which clustering had been zeroed by Illumina were manually clustered. Individuals with a call rate of >95% were retained in the analysis. Further quality control was performed in plink v1.07 (Purcell *et al*. 2007) with the following criteria: locus call rate >99%, minor allele frequency (MAF) >0.01 and deviation from Hardy–Weinberg Equilibrium (HWE) *P* > 1e-05.

Using the 5805 individuals and SNPs which had passed quality control, we next examined the distribution of MAF and the spacing between SNPs (positions were obtained from v3.1 of the sheep genome, http://www.livestockgenomics.csiro.au). We calculated linkage disequilibrium (LD) using the *r*^*2*^ statistic using all genotyped individuals in plink v1.07 (Purcell *et al*. 2007). For each SNP with MAF >0.05, *r*^*2*^ was calculated between the focal SNP and all SNPs with a MAF >0.05 which were <50 SNPs away within a 1000 Kb window.

### Construction of pedigrees and estimation of genomic relatedness

Two pedigrees (1 and 2) and a genomic relatedness matrix (GRM) were used in our analyses.

Pedigree 1: This pedigree was the most complete and accurate Soay sheep pedigree constructed using microsatellites to infer parentage. Maternities were assigned by field observation, and molecular parentage analysis was used to infer paternities (detailed description of methods in Morrissey *et al*. 2007). Individuals were genotyped at 14–18 microsatellite loci (Overall *et al*. 2005), and mean individual-level posterior support for paternity assignments was 98%. Parentage was assigned for all cohorts born between 1985 and 2009.

Pedigree 2: This pedigree was primarily built using molecular parentage analysis (for maternity and paternity) for all cohorts between 1980 and 2012. For each cohort maternity and paternity were inferred simultaneously using 315 high MAF, unlinked SNPs in the R package masterbayes (Hadfield *et al*. 2006) and all assignments were inferred with 100% confidence [see Table S1, Supporting information for a list of SNP names and map positions, more detailed information on how loci were selected can be found in (Johnston *et al*. 2013)]. For 96 of 3515 sheep with a mother previously assigned through observation, a different mother was found using SNP-based assignments (2.7%). Among these, about half were lambs found as dead neonates, indicating that in these cases, maternity is difficult to assign accurately in the field. For 2113 sheep with paternity assignments obtained from both Pedigree 1 and SNP-based inference, only 91 assignments differed (4.4%).

During the construction of Pedigree 2, not all parentage inferences could be made based on SNP genotypes alone, as we have not genotyped all offspring and their candidate parents (particularly for individuals alive prior to 1990). We used observations or assignments inferred using microsatellites to fill in the gaps. In 1257 cases where no maternity was assigned using molecular markers, field observational data were used. For 222 lambs without assigned fathers, paternity data from Pedigree 1 were used if confidence of assignment was >95%. For Pedigrees 1 and 2, pairwise relatedness between all individuals was estimated using the R package pedantics (Morrissey & Wilson 2010).

Genomic relatedness: The genomic relatedness between all pairs of SNP genotyped individuals was estimated in gcta v1.04 which estimates the proportion of the genome identity-by-state (IBS) between individuals. At each locus, relatedness was scaled by the expected heterozygosity 2*pq* (Yang *et al*. 2010, 2011a). No adjustments for sampling error or difference in allelic spectrum between genotyped SNPs and causal variants were made.

### Estimation of quantitative genetic parameters using univariate and bivariate models

As the genetic architecture of body size changes across ontogeny in Soay sheep (Wilson *et al*. 2007), the phenotype data set was split into four age classes: neonates, lambs, yearlings and adults. All analyses were run within each age class. Neonates were defined as individuals captured within 5 days of birth; only birth weight was available for this age class. Individuals were classified as lambs or yearlings if they had August phenotype data at age 4 months or 16 months, respectively, or were found post-mortem before age 14 months or 26 months, respectively. Adults were defined as individuals with August phenotype data at age 28 months or older or individuals with post-mortem measurements after age 26 months. Repeated measures within each age class only exist for adults with August phenotypes, but the same individual could occur in all four age classes.

Phenotypic variance for body size traits was partitioned into genetic and environmental variance components within each age class using animal models, which can fit both fixed and random effects. Fixed effects in the models differed between age classes. Fixed effects were chosen to mainly include effects with biologically or statistically relevant effects on the traits, especially those that are consistent across age classes. A detailed list of the fixed and nongenetic random effects fitted can be found in Table 1. All analyses were conducted in asreml-r (Gilmour *et al*. 2009).

**Table 1:** Fixed and nongenetic random effects fitted in the univariate animal models. Whether a term was fitted as a covariate or factor is shown in brackets (C or F, respectively)

	Fixed effects				Nongenetic random effects			
		
Traits	Neonates	Lambs	Yearlings	Adults	Neonates	Lambs	Yearlings	Adults
Weight	Sex, litter size, age at capture (days, F)	Sex, litter size, age at capture (months, F)	Sex	Sex, age at capture (years, F)	Birth year (F)	Birth year (F)	Birth year (F)	Birth year (F), permanent environment, year of capture (F)
Foreleg		Sex, litter size, age at capture (months, F)	Sex	Sex, age at capture (years, F)	Birth year (F)	Birth year (F)	Birth year (F)	Birth year (F), permanent environment, year of capture (F)
Hindleg		Sex, litter size, age at capture (months, F)	Sex	Sex, age at capture (years, F)	Birth year (F)	Birth year (F)	Birth year (F)	Birth year (F), permanent environment, year of capture (F)
Metacarpal length		Sex, litter size, age at death (months, C)	Sex	Sex, age at death (years, F)	Birth year (F)	Birth year (F)	Birth year (F)	Birth year (F)
Jaw length		Sex, litter size, age at death (months, C)	Sex	Sex, age at death (years, F)	Birth year (F)	Birth year (F)	Birth year (F)	Birth year (F)

We first analysed trait variance using the following univariate models:


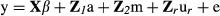
(Model1)


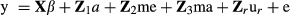
(Model2)

where **y** is the vector of phenotypic observations for all individuals, **X** is an incidence matrix linking individual records with vector of fixed effects *β;* **Z**_*1*_, **Z**_*2*_, **Z**_3_ and **Z**_*r*_ are incidence matrices which are used to relate random effects to the individual trait records. **a** is the vector of the additive genetic effects accounted by either pedigree or genomic relatedness, **m** is a vector containing maternal effects (with maternal environmental effects and maternal genetic effect not distinguished), **me** and **ma** are vectors containing the maternal environmental and maternal genetic effects, respectively, and **e** is a vector of residual effects. Additional random effects **u**_*r*_ varied between traits and are fitted with their own corresponding incidence matrix **Z**_*r*_. Birth year was fitted as a random effect in all models. For adult August phenotypic data, year of measurement and permanent environment effects were also fitted as random effects in all models. Models 1 and 2 were run using each of the three relatedness matrices (based on Pedigree 1, Pedigree 2 and GRM). Note that when maternal genetic effects were investigated in Model 2, the maternal relatedness matrix used was derived from the same source, for example, the Model 2 using genomic relatedness to detect direct genetic effects also used genomic data to detect maternal genetic effects. The variance explained by a random effect was expressed as a proportion of the total phenotypic variance for the trait after accounting for fixed effects in the model. The statistical significance of random effects was assessed using likelihood ratio tests (LRT) assuming a χ^2^ distribution with one degree of freedom.

We also estimated covariances between all five traits measured in adults using bivariate models, one for each of the ten trait combinations using each of the three relatedness matrices. Covariances were estimated using unstructured variance models. Fixed and random terms included in the models were identical to those included in univariate model 1 (see Table 1), except that as it accounted for very little variance (see univariate model results), birth year was not fitted. The nongenetic covariance between skeletal and August catch traits was captured with a permanent environment term, as residual variance structure was estimated using an *idh* term while fixing skeletal variance to near zero.

While the primary approach in this study is to compare the three different relatedness structures, as they arose in the Soay sheep study system, it is important to consider the separate effects of pedigree certainty, completeness and size. First, differences between Pedigree 1 and Pedigree 2 could potentially be explained by (i) the three additional cohorts included in Pedigree 2; and (ii) maternities which were inferred using genetic markers in Pedigree 2 leading to slightly larger sample sizes. To eliminate this possibility, we pruned the Pedigree 2 data set for each trait to only include individuals which were present in the Pedigree 1 data set and re-analysed the data using Model 1. Second, differences between Pedigree 2 and the genomic relatedness could arise due to the fact that the Pedigree 2 analyses include individuals with phenotype data which have not been SNP genotyped. We therefore pruned the data set analysed with Pedigree 2 to include only SNP genotyped individuals and reran univariate analyses with Model 1 to check whether this made any difference to the results obtained.

Finally, we explored whether the heritability captured by SNPs correlates with the number of SNPs used to estimate relatedness. In total, 37 037 autosomal SNP loci were used to estimate genomic relatedness. We randomly sampled 2.5%, 5%, 10%, 30%, 50%, 70% and 90% of the available markers, corresponding to 926, 1852, 3704, 11 111, 18 518, 25 926 and 33 333 autosomal markers, respectively. This procedure was replicated 50 times, and adult traits were analysed with each resulting genomic relatedness matrix using Model 1.

## Results

### Marker information

**Fig. 1:**
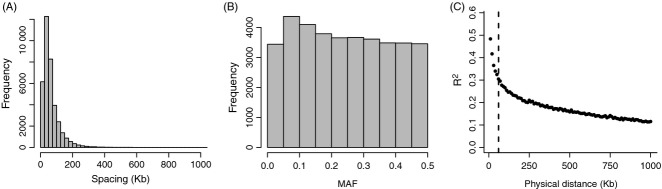
Summary of SNP characteristics. Shown are (A) the distribution of spacing between adjacent SNP markers, (B) the distribution of minor allele frequencies at SNP markers and (C) the decay of LD against physical distance between SNPs. Each closed circle shows the mean LD within a 10 Kb window. The dashed vertical line indicates the median spacing between adjacent SNP markers.

During quality control, 2547 SNPs were removed because of low call rate, 10 521 SNPs were removed because of low MAF, and 580 SNPs were removed because of deviation from Hardy–Weinberg equilibrium. The resulting data set consisted of 37 037 informative autosomal SNPs. Mean and median spacing were 65.9 and 50.2 Kb, respectively (Fig. 1A). Mean and median MAF were both 0.24, and the MAF distribution was relatively uniform (Fig. 1B). LD decayed with distance; at an intermarker distance of 50–60 Kb, which roughly corresponds to the average spacing between adjacent SNPs, mean *r*^*2*^ was 0.30, but *r*^*2*^ dropped to 0.11 at an interlocus distance of 1 Mb (Fig. 1C).

### Comparison of pedigree and relatedness estimates

Compared to Pedigree 1, Pedigree 2 contained substantially more information (Table 2), even when taking into account that cohorts 2010–2012 were included in Pedigree 2, but not Pedigree 1. While the number of maternity assignments increased by more than a third, maternal assignment rates in nonfounders stayed approximately equal (95% in Pedigree 1 vs. 94% in Pedigree 2). The number of paternity assignments more than doubled, and paternal assignment rates in nonfounders increased from 49% to 72%. The number of pairwise full-sibs almost tripled (Table 2), and as a result of the improved parentage information, Pedigree 2 contained much more grandparental information and considerably more paternal sibs.

**Table 2:** Comparison of summary statistics between Pedigree 1 and Pedigree 2. All pedigree statistics were obtained using the R package pedantics (Morrissey & Wilson 2010)

	Pedigree 1	Pedigree 2
Records	5068	6740
Maternities	4373	5981
Paternities	2253	4593
Full-sibs	129	349
Maternal sibs	13496	19913
Maternal half-sibs	13367	19564
Paternal sibs	13580	48487
Paternal half-sibs	13451	48138
Maternal grandmothers	3122	4917
Maternal grandfathers	1893	4031
Paternal grandmothers	1149	2734
Paternal grandfathers	946	2917
Maximum pedigree depth	9	10
Founders	478	404
Mean maternal sibship size	4.579	4.528
Mean paternal sibship size	4.308	6.309
Nonzero *F*	120	813
*F* > 0.125	8	27
Mean pairwise relatedness	0.00295	0.00587
Pairwise relatedness >=0.125	0.00905	0.01434
Pairwise relatedness >=0.25	0.00322	0.00421
Pairwise relatedness >=0.5	0.00053	0.00048

As a result of the changes, mean relatedness was higher in Pedigree 2 (6.4 × 10^−3^) than in Pedigree 1 (3.1 × 10^−3^) or using genomic relatedness (−1.7 × 10^−4^), but the variance in relatedness increased from Pedigree 1 (4.5 × 10^−4^) to Pedigree 2 (6.6 × 10^−4^) to genomic relatedness (1.3 × 10^−3^, Table S3, Supporting information).

The three methods (Pedigree 1, Pedigree 2 and genomic relatedness) generated correlated pairwise relatedness estimates. Pedigree 1 relatedness correlated with Pedigree 2 relatedness (intercept = −3.1 × 10^−3^, slope = 0.96, *R*^2^ = 0.59, *P* < 0.0001, Fig. S1, Supporting information) and genomic relatedness (intercept = −3.4 × 10^−3^, slope = 0.92, *R*^2^ = 0.32, *P* < 0.0001). Pedigree 2 relatedness correlated with genomic relatedness estimated using SNPs (linear regression, intercept = −7.4 × 10^−3^, slope = 0.90, *R*^2^ = 0.51, *P* < 0.0001).

The differences in information of the two pedigrees was retained when considering only informative individuals for univariate trait analyses presented in this study (Table S2, Supporting information). While the total number of individuals decreased compared with the full pedigrees, Pedigree 2 still contained much more information than Pedigree 1 across all statistics, both for the estimation of direct (Table S2) and maternal genetic effects (Table S3) in all traits and age classes.

### Comparison of variance components estimated using pedigree or marker relatedness

**Fig. 2:**
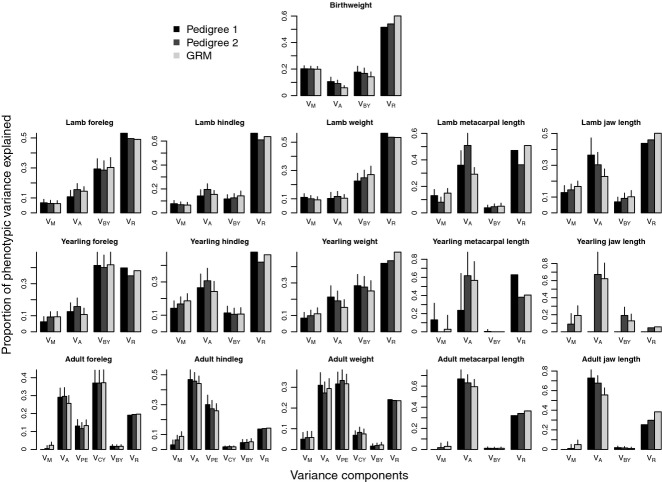
Comparison of variance components from univariate animal models of body size using Model 1. Results are shown from top to bottom for neonates, lambs, yearlings and adults. Variance components differed between models, and shown are maternal effect (V_M_), additive genetic effect (V_A_), birth year effect (V_BY_), measurement year effect (V_CY_), permanent environment effect (V_PE_) and the residual variance (V_R_). Error bars indicate the standard error of the estimates.

**Fig. 3:**
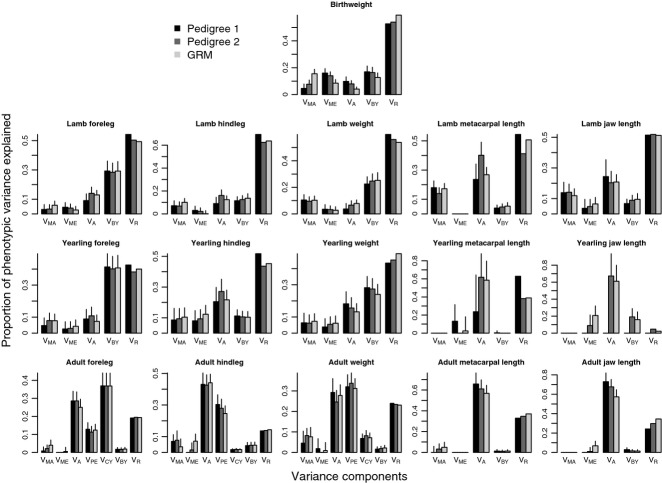
Comparison of variance components from univariate animal models of body size using Model 2. Results are shown from top to bottom for neonates, lambs, yearlings and adults. Variance components differed between models, and shown are maternal genetic effect (V_MA_), maternal environment effect (V_ME_), additive genetic effect (V_A_), birth year effect (V_BY_), measurement year effect (V_CY_), permanent environment effect (V_PE_) and the residual variance (V_R_). Error bars indicate the standard error of the estimates.

Sample sizes (total number of observations, number of known maternities, and for adults, number of unique individuals) for all univariate models are shown in Table 3. Estimates for all the variance components in Model 1 and Model 2 analyses are shown in Figs 2 and 3 and Tables S4 and S5, respectively.

**Table 3:** Comparison of sample sizes of animal models using the pedigrees and genomic relatedness. For adult August catch traits where repeated measures are available, the total number of observations is shown in brackets

		Pedigree 1		Pedigree 2		Genomic relatedness	
				
Age class	Trait	*N*	*N* unique maternities	*N*	*N* unique maternities	*N*	*N* unique maternities
Neonates	Birthweight	3182	801	3648	909	3181	808
Lambs	Foreleg	1544	565	1804	651	1726	627
	Hindleg	1608	568	1868	654	1791	631
	Weight	1702	604	1965	690	1849	662
	Metacarpal length	1074	501	1331	609	1298	601
	Jaw length	1207	563	1468	670	1349	616
Yearlings	Foreleg	749	393	823	428	792	420
	Hindleg	774	399	850	434	817	425
	Weight	789	403	869	439	831	429
	Metacarpal length	195	161	227	188	219	182
	Jaw length	247	202	281	229	253	211
Adults	Foreleg	803 (2247)	417	877 (2435)	443	855 (2375)	432
	Hindleg	816 (2345)	420	891 (2542)	447	867 (2477)	435
	Weight	813 (2364)	418	889 (2564)	445	865 (2499)	433
	Metacarpal length	595	358	643	373	621	364
	Jaw length	639	372	692	389	661	375

#### Neonates

In Model 1, variance due to maternal effects and heritability of birth weight decreased from Pedigree 1 to Pedigree 2 to genomic relatedness (Table S4, Supporting information Fig. 2).

In Model 2, estimates of maternal genetic effects increased from Pedigree 1 to Pedigree 2 to genomic relatedness, while maternal environmental effects and heritability decreased from Pedigree 1 to Pedigree 2 to genomic relatedness (Table S5, Supporting information Fig. 3).

#### Lambs

In Model 1, heritability for the August traits (foreleg, hindleg, weight) generally increased from Pedigree 1 to genomic relatedness to Pedigree 2 (Table S4, Fig. 2), but a less obvious pattern was observed in the skeletal traits. In metacarpal length, Pedigree 2 estimated substantially larger heritabilities than either Pedigree 1 or genomic relatedness, while in jawbone, heritability was highest using Pedigree 1. Variance due to maternal effects in the August catch traits increased from Pedigree 1 to Pedigree 2 to genomic relatedness, while in jawbone, the opposite pattern was observed.

In Model 2, when dissecting maternal effects into maternal genetic and maternal environmental components, all estimates of relatedness allowed detection of maternal genetic effects in three traits (weight, jawbone and metacarpal length), while maternal genetic effects were only significant in foreleg length and hindleg length in models using genomic relatedness. For hindleg length and weight, heritabilities estimated using Pedigree 1 were not significant when a maternal genetic effect was fitted (Table S5, Fig. 3). No significant maternal environmental effects were found in any of the traits.

#### Yearlings

In yearlings, heritabilities were higher than in lambs (Table S4, Fig. 2). Maternal effects increased from Pedigree 1 to Pedigree 2 to genomic relatedness. No significant maternal effects were found in any of the models analysing jawbone or metacarpal length, which is likely to be partly explained by the low sample sizes for the skeletal traits (Table 3, also see Standard errors around the estimates, Table S3).

Using Model 2, despite the strong maternal effects in the August traits in Model 1, maternal genetic effects were only found in foreleg length, while maternal environment effects were only observed in hindleg length, both of which were only significant in models using genomic relatedness (Table S5, Fig. 3). Neither Model 1 nor Model 2 analysing jawbone converged when using Pedigree 1 (Tables S4 and S5), presumably due to lack of data (Table 3).

#### Adults

In adults using Model 1, heritabilities were high, ranging between 0.25 and 0.73 (Table S4, Fig. 2) with the highest estimates observed in the two skeletal traits. Maternal effect estimates in all traits increased from Pedigree 1 to Pedigree 2 to genomic relatedness (Table S4, Fig. 2). When separating maternal effects into maternal genetic and maternal environmental effects (Model 2), most of the maternal variance was attributed to maternal genetic effects, but no significant maternal effects (genetic or environmental) were found (Table S4, Fig. 3).

Accuracy of maternal effect, maternal genetic effect and heritability estimates, either the relationship between estimate and standard error, or standard error alone, generally increased from models using Pedigree 1 to Pedigree 2 to models using genomic relatedness, a pattern which emerged relatively systematically across all four age classes (Table S5).

### Genetic and environmental covariances among traits

**Fig. 4:**
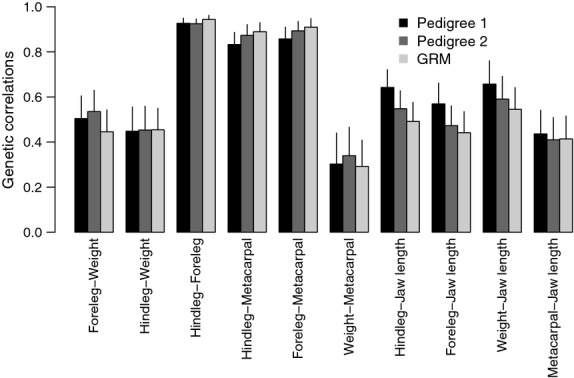
Estimates of genetic correlations from bivariate animal models of adult body size using Model 1. Error bars indicate the standard error of the estimates.

All genetic covariances were positive (Table S6, Supporting information), and resulting genetic correlations were between 0.29 and 0.94. Genetic correlations were, as might be expected, strongest among leg length measures (Table S6, Fig 4) and weakest between leg length measures and weight, with trait combinations involving jawbone being somewhat intermediate in magnitude. Genetic covariances and correlations obtained using the three relatedness estimates were very similar, with substantially overlapping standard errors, the only exception being genetic correlations involving jawbone length which tended to decline with improving genetic information. There was a fairly consistent pattern of genetic covariances decreasing with improving relatedness information (Table S6), but a much less consistent pattern was observed for genetic correlations. The majority of estimates of maternal covariance were positive, but some of the maternal covariances estimated using Pedigree 1 were weakly negative. In this data set, interpreting maternal covariances and correlations should be performed with extreme caution, as in most cases variance attributed to maternal effects was not significant in the univariate analyses (Table S4). Only hindleg length and weight showed significant maternal effects when using either Pedigree 2 or the GRM, and between these two traits, maternal correlations were very strong (0.916 and 0.936, respectively, Table S6). The standard errors of genetic covariances and correlations were generally very small compared with the estimates, and differed little between the three different estimators, tending to be smaller for pedigree 2 and the GRM. Estimates of covariances and correlations for all other random effects can be found in Table S6.

### Comparisons using Pedigree 2 pruned to only include those animals present in Pedigree 1 or genomic relatedness analyses

Pedigree 2 heritability estimates retaining only the individuals used in the Pedigree 1 analysis did not differ much from the Pedigree 2 estimates where all individuals were used (Table 4) and were generally closer to Pedigree 2 estimates than to the Pedigree 1 estimates. Standard errors were intermediate to those observed in the Pedigree 1 and Pedigree 2 analyses. This suggests that the increased sample sizes in the Pedigree 2 analyses as a result of both three additional cohorts and SNP-derived maternities on its own cannot explain the differences between Pedigree 1 and Pedigree 2 estimates.

**Table 4:** Estimates of V_A_/V_P_ for body size traits fitting pedigree or genomic relatedness individually. Table shows mean and standard error estimates from univariate models. Shown are estimates obtained using Pedigree 1, Pedigree 2, Pedigree 2 only including individuals included in the Pedigree 1 analysis (Pedigree 2_PED1_), Pedigree 2 only including individuals with genotype data (Pedigree 2_SNP_) and genomic relatedness (GRM)

Age class	Trait	Pedigree 1	Pedigree 2	Pedigree 2_PED 1_	Pedigree 2_SNP_	GRM
Neonates	Birthweight	0.106 (0.034)	0.091 (0.026)	0.083 (0.027)	0.086 (0.026)	0.059 (0.017)
Lambs	Foreleg	0.108 (0.043)	0.155 (0.041)	0.127 (0.042)	0.149 (0.04)	0.145 (0.031)
	Hindleg	0.141 (0.052)	0.196 (0.048)	0.17 (0.05)	0.193 (0.047)	0.155 (0.033)
	Weight	0.102 (0.044)	0.116 (0.036)	0.098 (0.037)	0.109 (0.034)	0.104 (0.026)
	Metacarpal length	0.36 (0.11)	0.509 (0.092)	0.524 (0.103)	0.514 (0.092)	0.292 (0.051)
	Jaw length	0.364 (0.108)	0.303 (0.078)	0.312 (0.09)	0.313 (0.079)	0.23 (0.047)
Yearlings	Foreleg	0.126 (0.055)	0.157 (0.054)	0.15 (0.054)	0.15 (0.052)	0.108 (0.04)
	Hindleg	0.266 (0.083)	0.307 (0.075)	0.296 (0.078)	0.313 (0.077)	0.243 (0.061)
	Weight	0.213 (0.069)	0.19 (0.061)	0.162 (0.063)	0.208 (0.064)	0.15 (0.048)
Adults	Foreleg	0.291 (0.052)	0.296 (0.049)	0.286 (0.05)	0.289 (0.049)	0.257 (0.044)
	Hindleg	0.468 (0.065)	0.458 (0.058)	0.446 (0.062)	0.448 (0.059)	0.441 (0.051)
	Weight	0.31 (0.061)	0.273 (0.054)	0.267 (0.056)	0.271 (0.055)	0.294 (0.048)
	Metacarpal length	0.668 (0.086)	0.631 (0.078)	0.608 (0.085)	0.644 (0.08)	0.594 (0.07)
	Jaw length	0.729 (0.084)	0.677 (0.076)	0.685 (0.081)	0.677 (0.079)	0.556 (0.072)

Pedigree 2 heritability estimates using the subset of SNP genotyped individuals were very close to estimates using all individuals with Pedigree 2 information (Table 4), indicating that the small difference in data sets shown in Figs 2 and 3 is unlikely to be a major explanation for the systematic differences between genomic heritability and pedigree heritability.

### Number of markers required to estimate heritability

**Fig. 5:**
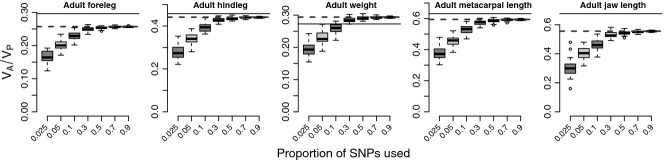
Estimated heritability of adult body size as a function of increasing marker number. Box and whiskers show the median and spread of 50 replicate sampled sets of SNPs. The solid and dashed lines represent Pedigree 2 heritability and genomic heritability estimates using all available markers, respectively.

For all five proxies of adult body size, genomic heritability estimates increased with increasing number of markers, but asymptoted at around 50% of the total number of informative SNPs (*n* = 18 518), suggesting that adding more loci to our current panel will not necessarily lead to improved genomic heritability estimates (Fig. 5). The spread between sampled subsets of SNPs decreased with increasing number of markers.

## Discussion

Our results show that substantial pedigree improvements did not lead to large changes in heritability estimates or genetic correlations. Most of the genetic variance as estimated by the best pedigree is accounted for by genomewide SNP markers, and genetic correlations among traits were comparable with those obtained using the best available pedigree. The proportion of genetic variance captured by genomic markers increased with marker density, but increasing density beyond the capacity offered by the OvineSNP50 chip will probably only lead to marginal improvements in heritability estimation. Superior estimates of relatedness resulted in both higher general maternal effects and maternal genetic effects. We also show that standard errors of heritability and maternal effects decrease when more accurate estimates of relatedness are used, resulting in increased power to detect significant effects. Taken together, the above results are encouraging for the usage of dense marker panels to address quantitative genetic questions in wild populations, including those for which pedigrees are unobtainable.

### Effects of improved pedigree information on heritability estimates

Our results confirm that all proxies of body size have a solid genetic basis and are genetically correlated in a wild population of Soay sheep. Skeletal traits which were measured *post-mortem* showed higher heritability than traits measured on live sheep, and leg length showed higher heritabilities than body weight, which is consistent with previous findings (Beraldi *et al*. 2007; Wilson *et al*. 2007). In line with earlier results (Wilson *et al*. 2007), heritability estimates increased with age for all traits. Despite the addition of several cohorts of data as previously published heritability estimates in the same population, heritability estimates in this study are surprisingly consistent with previous papers (Wilson *et al*. 2005, 2007; Beraldi *et al*. 2007). In addition, even with tremendous pedigree improvements, heritability estimates of body size remain relatively unchanged across four age classes between Pedigree 1 and the improved Pedigree 2. We also show that only a small proportion of the differences in estimates between Pedigree 1 and Pedigree 2 can be attributed to the larger sample sizes in the latter, meaning that pedigree improvements are mainly responsible for the observed difference in performance.

While some differences in estimates of heritability and genetic correlations were observed, standard errors generally overlapped indicating that differences were minimal. In addition, we observed no consistent downwards or upwards bias in estimates from Pedigree 1 compared with those obtained with the superior Pedigree 2. Our results are in line with other results from other unmanaged populations, which suggest that pedigree errors within the range most commonly accepted in natural populations (5–20%) lead to heritability estimates virtually indistinguishable from those estimated using improved pedigrees (Charmantier & Reale 2005; Morrissey *et al*. 2007), and with simulation results in our study population which showed that estimates of genetic correlations are robust to pedigree errors within the range typical in natural populations (Morrissey *et al*. 2007). Results obtained in natural populations, including ours, are in contrast with results obtained in animal breeding, where pedigree errors led to drastic downwards bias in heritabilities or breeding values in some studies (Lee & Pollak 1997; Banos *et al*. 2001), but not in others (Israel & Weller 2000; Clement *et al*. 2001). There are several potential explanations for the contrasting findings between livestock and unmanaged populations. First, while pedigree structure varies tremendously between different taxa, it is generally much more heterogeneous and connected in wild populations. In livestock, sibships are generally larger than is commonly observed in wild populations, sometimes differing in orders of magnitude [e.g. (Lee & Pollak 1997)]. As a consequence of this, a single pedigree error may affect a much more substantial proportion of the population in livestock than in wild pedigrees. Second, while pedigree errors are often introduced at random in livestock simulations (Lee & Pollak 1997; Banos *et al*. 2001), in wild populations, misassigned parents may be nonrandomly sampled from the pool of candidate parents, both with respect to genotype and shared environment. Erroneously assigned parents are potentially closely related to the true parents, thereby damping the downward bias in heritability estimates as a result of those errors. Pedigree structure (i.e. connectivity, depth, sibship sizes, reproductive skew), data structure (i.e. sources of common environment, relatedness between phenotyped individuals, number of individuals with recorded phenotypes), trait heritability, and the presence of a systematic pattern in parentage errors may all affect the severity of the bias in heritability estimates as a consequence of misassignments. However, the effects on heritability, and particularly genetic correlations, are poorly understood. Hence, we believe that there is a definite need to explore the relative importance of these effects, both by simulations and by empirical work in a wide range of taxa before we can be confident whether the patterns we see in both the blue tit (Charmantier & Reale 2005) and Soay sheep (Morrissey *et al*. 2007) data sets are anomalies or indicative of a general phenomenon.

### Maternal (genetic) effects for body size

Maternal effects represent important environmental or genetic sources of phenotypic variation (Rasanen & Kruuk 2007) and failure to account for them can lead to inflated heritability estimates (Kruuk & Hadfield 2007). We confirm previous findings of maternal effects in neonatal and lamb traits (Wilson *et al*. 2005), but we also show that maternal effects are important in older age classes. Maternal effects are potentially confounded with direct genetic effects, with the extent of confounding dependent on the completeness of parentage information and type of relatedness information present. The way in which pedigree errors bias maternal effects and confound heritability and maternal effects have not been well documented (Morrissey *et al*. 2007), possibly because not all pedigree structures lend themselves to explore this. Here, we have demonstrated that the improved accuracy and completeness of Pedigree 2 and the even more accurate estimates of relatedness at genomic markers did have a positive effect on power to detect maternal effects.

When maternal effects were partitioned into genetic and environmental components, maternal genetic effects were found in all neonatal and lamb traits, and for foreleg in yearlings. While maternal genetic effects were not statistically significant in the remaining traits and ages, they explained much more of the phenotypic variance than the maternal environment in yearlings and adults. We again observed that more accurate estimates of relatedness led to higher and more precise maternal genetic effect estimates (Table S5). In addition, direct and maternal effects were less confounded with each other when using the GRM compared with Pedigree 2, and with Pedigree 2 compared to Pedigree 1; as heritability decreased less in models where a maternal genetic effect was fitted compared with models where only a general maternal effect was fitted. The amount of phenotypic variance explained by maternal genes is not only of statistical significance, as maternal genotype explains up to three times as much of the phenotypic variance as genes carried by the phenotyped individuals themselves (Table S5). Our results confirm our suspicion that poor estimates of relatedness are a major explanation for why maternal genetic effects are rarely found in natural populations. Using SNP-derived relatedness estimates in a quantitative genetic framework may therefore lead to a considerable reappraisal of the importance of maternal genetic effects and thus a better understanding of micro-evolutionary trends (or the lack thereof) in wild populations (Larsson *et al*. 1998; Kruuk *et al*. 2001).

### Standard errors

A systematic effect of improvements in the estimation of relatedness was observed in standard errors of heritability, (general) maternal effects and maternal genetic effects. Standard errors generally declined from Pedigree 1 to Pedigree 2 to the GRM, suggesting that more accurate estimates of relatedness allow the estimation of variance components with more precision. Only a minor proportion of the differences can be explained by differences in sample size between the data sets, indicating that the smaller standard errors are a direct result of improvements in relatedness estimates. This suggests that improved relatedness estimates can increase power to detect significant quantitative genetic parameters.

### Comparison of pedigree and genomic relationship information

On average, genomic relatedness accounted for 84% of the genetic variance, meaning that most of the heritability as estimated using Pedigree 2 is captured by SNP markers on a 50K SNP chip. We also show that the difference in heritability estimates between Pedigree 2 and the GRM cannot be explained by the small differences in data set size and composition between the two analyses. The proportion of additive genetic variance explained by genomic markers is much higher than is found in human populations when unrelated individuals are used (Yang *et al*. 2010, 2011b), but comparable with cattle data (Jensen *et al*. 2012; Haile-Mariam *et al*. 2013). Similar to cattle, linkage disequilibrium in Soay sheep is high due to a low effective population size (Kijas *et al*. 2012). The high LD in combination with the presence of close relatives in the data set leads to alleles at causal loci being predicted relatively well by alleles at genotyped SNPs. As the probability of tagging all causal mutations is a function of linkage disequilibrium between genotyped SNPs and unobserved QTL, and thus indirectly marker density, using a larger number of markers could potentially explain more of the genetic variance.

Genetic variance captured by SNPs does increase with marker density, but asymptotes at around half the total number of polymorphic markers available to us, suggesting that adding more common SNP markers is not expected to capture all of the pedigree heritability (Fig. 5). One possible explanation for this gap is that our pedigree heritability estimates are inflated. Even though we have included common environmental effects in our models (e.g. birth year, measurement year) to account for environmental sources of covariance, these may not accurately capture the fine-scaled spatio-temporal heterogeneity covarying with phenotypic variance. Relatives often share habitats and the colinearity between relatedness and shared environmental conditions may potentially lead to upwards bias in heritability estimates (Van Der Jeugd & McCleery 2002; Stopher *et al*. 2012). However, shared environment and genomic relatedness would be expected to be similarly confounded, meaning that this cannot explain the systematically lower heritability estimates obtained with the SNPs. One way of potentially avoiding bias introduced by shared environment is adopting a strategy similar to Yang *et al*. (2010) where heritability is estimated using SNP markers in a subset of unrelated individuals. This is not feasible in this population, as due to the highly connected pedigree structure most individuals are related to other individuals. By pruning the data set through the exclusion of individuals which are related to any other individuals above 0.025 as recommended in gcta (Yang *et al*. 2011a), resulting sample sizes in this data set are too small to conduct any meaningful analyses. Another explanation for the difference in heritability estimates between Pedigree 2 and the GRM is that causal loci are in imperfect LD with genotyped SNPs. Body size is associated with fitness in many organisms (Blanckenhorn 2000), including Soay sheep (Coltman *et al*. 2001). Selection will generally cause minor allele frequencies at causal loci to be lower than those at genotyped SNPs, and thus to be poorly tagged by common SNP markers. In contrast, the pedigree estimates the probability of IBD at both common and rare loci. The pattern observed in cattle, where a substantially lower proportion of genetic variance is captured in fitness traits than in production traits (Jensen *et al*. 2012; Haile-Mariam *et al*. 2013) is in line with this argument.

We also show that, using relatedness from a dense SNP panel, we are able to estimate genetic correlations between body size traits which are comparable in magnitude and precision with estimates obtained using both Pedigree 1 and the superior Pedigree 2. The genetic correlations between hindleg length and weight measured here (at around 0.45) are similar to previous within-male estimates obtained using Pedigree 1 (0.50, Morrissey *et al*. 2012). Perhaps surprisingly, genetic correlations were substantially lower than earlier estimates from the same population [between 0.74 and 0.8 (Coltman *et al*. 2001; Milner *et al*. 2000)]. While this difference may partly be explained by the different modelling approaches adopted [here: combined sex models; previously published results: sex-specific modelling (Coltman *et al*. 2001; Milner *et al*. 2000; Morrissey *et al*. 2012)], we believe that there are two more plausible explanations for this difference. First, Pedigree 1 (constructed by and used in Morrissey *et al*. (2012)) was a major improvement, both in size and in error rate, over the pedigrees used in the earlier papers (Milner *et al*. 2000; Coltman *et al*. 2001). Thus, it is conceivable that, in earlier analyses, genetic covariances and correlations were upwardly biased due to confounding with the maternal covariance structure. In support of this explanation, genetic covariances decreased relatively consistently with improving relatedness information in our analyses. This is also consistent with a study conducted in a natural population of passerine birds, which showed that genetic correlations estimated using parent–offspring regressions were systematically higher than genetic correlations estimated using an animal model, which should suffer less from bias due to shared environment (Åkesson *et al*. 2008). Second, the data set differed between the various papers with respect to the age classes which were included (here: adults only, Morrissey *et al*. 2012: adults and yearlings, Coltman *et al*. 2001; Milner *et al*. 2000: adults, yearlings and lambs). Possibly as a result of this, V_A_ for weight was much lower in previous analyses. And as correlations are calculated by scaling the covariance by the variances, this could partly explain the lower estimates for the genetic correlation. Indeed, the genetic covariances estimated in Coltman *et al*. (2001, 3.2 and 4.1 for males and females, respectively) were very similar to our estimates (ranging between 3.1 and 3.3), although the estimates from Milner *et al*. (2000) were substantially higher than ours (5.5 and 4.5 for males and females, respectively).

Another consistent pattern which emerged from our results is that genetic covariances and correlations decreased with increasingly accurate estimates of relatedness when jaw length was involved in the analysis (see Fig. 4). The proximate explanation for this is that the estimated genetic variance for jaw length differed between the various relatedness estimates in a similar manner (see Table S4), but we do not yet understand why this pattern occurs in the data. In the field of human genetics, estimating genetic correlations using genomic relatedness has seen a slower uptake than estimating heritability (Lee *et al*. 2012; Vattikuti *et al*. 2012) and we are not aware of any examples in natural populations. As natural selection acts simultaneously on multiple traits, and multivariate analyses are often required to understand the potential response to selection (Lande & Arnold 1983; Blows 2007; Kruuk *et al*. 2008), these are highly encouraging results for future studies.

### Prospects for genomic-relatedness-based quantitative genetics in natural populations

To date, estimating quantitative genetic parameters such as heritability and genetic correlations in the wild has generally been limited to systems where it is possible to reconstruct pedigrees. Using marker-based methods to infer relationships between individuals could potentially allow the estimation of heritability in systems where reconstructing pedigrees or sibships is not practical or feasible (Ritland 1996). Attempts have been made using microsatellite markers, but estimates were often shown to be wildly different from those obtained using pedigrees (Thomas *et al*. 2002; Garant & Kruuk 2005). An explanation for this is that even in structured populations, the mean relatedness is typically low with little variance, and the imprecision with which relationships are inferred using low-density marker data (Csilléry *et al*. 2006). We clearly demonstrate that heritability estimates obtained from dense SNP data are in correspondence with pedigree estimates. Our results confirm recent work on a pedigreed population of great tits (Robinson *et al*. 2013; Santure *et al*. 2013) where heritability as estimated using genomic relatedness at SNP markers was similar to pedigree heritability. However, to achieve this, these studies relied on shrinking the genomic relatedness matrix by regressing marker relatedness towards pedigree relatedness following (Goddard *et al*. 2011), adjusting for sampling error as a result of the finite number of markers used. Therefore, their results are not directly comparable with methods such as used here and elsewhere (Yang *et al*. 2010), where only the genetic variance captured by genotyped SNPs is estimated.

Our results are encouraging for those aiming to estimate quantitative genetic parameters in a natural population without a pedigree. But we argue that, for several reasons, scientists need to carefully consider if density and genomic coverage of their marker panel are appropriate to estimate heritability in their study system. First, estimation of relationships is subject to sampling error, and generally more markers should lead to more precise estimates of relatedness. Marker number should ideally be larger than the number of individuals to avoid singularities in the relatedness matrix (Van Raden 2008). Nonsingular matrices can be obtained when marker number is low using weighted relatedness matrices following (Van Raden 2008) or using gcta (Yang *et al*. 2010, 2011a). Second, the extent of LD, for example, due to historical effective population size, dictates how well SNP loci tag causal loci (Hayes & Goddard 2010; Gay *et al*. 2013). In systems such as the Soay sheep where the effective population size is low, LD is high and a relatively modest number of markers is enough to capture most of the genetic variance. However, in other systems such as passerine birds, effective population sizes can be very large, and thus much denser marker panels are needed. For example, it has been shown that currently available SNP chips do not sufficiently tag the genome to be a substitute for pedigrees in the estimation of heritability of body size in a long-term study of great tits (Robinson *et al*. 2013), possibly as a result of very low LD (Van Bers *et al*. 2012). Third, ideally within-population variation in relatedness should be present. As individuals are more distantly related, genomic heritability reflects the covariance between phenotypic resemblance and genomic similarity at the genotyped SNPs only and can thus be considerably lower than pedigree heritability, such as is found in human genetics (Yang *et al*. 2010). When data sets consist of both related and unrelated individuals, such as in the current study and, for example, studies using pedigreed cattle (Haile-Mariam *et al*. 2013), genotyped SNPs tag the whole genomic relationship including loci on different chromosomes, thereby leading to genomic heritability estimates closer to pedigree heritability (Zaitlen *et al*. 2013). It may not always be feasible to acquire the necessary knowledge concerning LD properties before having to settle for a certain number of markers, without doubt limited by financial constraints. However, we advocate that researchers make an educated guess whether thousands, tens of thousands or millions of markers are needed by taking into account both past (i.e. admixture events, bottlenecks, historical population size) and current (i.e. population structure, isolation from other populations) demographic processes which may have shaped their study population.

In addition to the aforementioned concerns, there is one final caveat to the applicability of dense genomic markers in estimating heritability in the field. Even if marker density is high enough to allow the estimation of heritability with an acceptable level of precision, there is still a need to account for confounding common environment effects. Just as when using a pedigree to estimate heritability, failure to identify and then include those sources of confounding in the models will inevitably lead to biased estimates (Kruuk 2004; Kruuk & Hadfield 2007). Maternal effects are an important example of such shared environment effects, especially for juvenile traits (Rasanen & Kruuk 2007). While it is possible to detect sibships using software such as colony2 (Wang & Santure 2009), identifying which sibs share maternal (rather than paternal) links may prove difficult without thorough field observational data or complete sampling of candidate parents.

In conclusion, we have demonstrated that in a free-living population of sheep, heritability estimates of body size traits and genetic correlations among traits did not change substantially despite radical improvements in pedigree quality. It is conceivable that improved relatedness information, either by pedigree or by genomic methods, can potentially have larger effects on heritability estimates in other traits and systems, if existing heritability estimates are less precise than is the case for body size in Soay sheep. Larger effects of improved relatedness estimates were seen when estimating maternal (genetic) effects. Furthermore, we have shown that in this population, most of the genetic variance and covariance as estimated by the pedigree is captured by SNPs on a commercially available 50K SNP chip. Increasing the number of markers is unlikely to yield improvements in the proportion of genetic variance which can be explained by SNPs. We suggest that dense marker panels can be used successfully to estimate quantitative genetic parameters in wild populations, but only if researchers (i) ensure that marker density in relation to levels of LD are high enough to tag causal variants; and (ii) account for sources of common environment in their models to avoid upwards bias of heritability estimates.

## Data accessibility

Phenotype data, pedigree data, SNP genotype data, inverse relatedness matrices and list of allele frequencies for SNP markers can be found on Dryad: Dryad entry doi:10.5061/dryad.367s2.

## Supporting information

**Table S1** List of SNP names used to infer parentage in the Pedigree 2 analysis. **Table S2** Summary statistics of pruned pedigrees for all univariate models 1. **Table S3** Summary statistics of pruned pedigrees for maternal genetic effect in all univariate models 2. **Table S4** Estimates of variance components  and their ratios to phenotypic variance  for model 1. **Table S5** Estimates of variance components  and their ratios to phenotypic variance  for model 2. **Table S6** Estimates of genetic and environmental covariance between adult body size traits. **Fig. S1** Correlations between pairwise relatedness estimates obtained using pedigree 1, pedigree 2 and genomic relatedness.
