# Supplementary material for: Estimating quantitative genetic parameters in wild populations: a comparison of pedigree and genomic approaches
Source: Mol Ecol. 2014 Jun 26;23(14):3434–51. doi: 10.1111/mec.12827 (PMC4149785; doi:10.1111/mec.12827)

**Table S1:** List of SNP names used to infer parentage in the Pedigree 2 analysis

| **Chromosome** | **OvineSNP50 name** | **Physical position** |
| --- | --- | --- |
| 1 | OAR1_1447599.1 | 1447599 |
| 1 | s15090.1 | 4239142 |
| 1 | s61691.1 | 18117744 |
| 1 | s40222.1 | 19034647 |
| 1 | OAR1_30388117.1 | 30388117 |
| 1 | s20982.1 | 32313144 |
| 1 | OAR1_40116778.1 | 40116778 |
| 1 | OAR1_47377110.1 | 47377110 |
| 1 | OAR1_60012212.1 | 60012212 |
| 1 | OAR1_78275547.1 | 78275547 |
| 1 | s68056.1 | 90937254 |
| 1 | s42405.1 | 102924875 |
| 1 | OAR1_107354355.1 | 107354355 |
| 1 | OAR1_130129851.1 | 130129851 |
| 1 | OAR1_149346875.1 | 149346875 |
| 1 | DU499024_430.1 | 153973394 |
| 1 | OAR1_160350281.1 | 160350281 |
| 1 | OAR1_171775698.1 | 171775698 |
| 1 | OAR1_188056499.1 | 188056499 |
| 1 | OAR1_212510864.1 | 212510864 |
| 1 | OAR1_236673559.1 | 236673559 |
| 1 | OAR1_253349819.1 | 253349819 |
| 1 | OAR1_266908731.1 | 266908731 |
| 1 | OAR1_282017475.1 | 282017475 |
| 1 | OAR1_287150413.1 | 287150413 |
| 1 | OAR1_291859755.1 | 291859755 |
| 1 | s38242.1 | 294180387 |
| 1 | s41901.1 | 298344733 |
| 2 | s06085.1 | 606868 |
| 2 | OAR2_4134550.1 | 4134550 |
| 2 | s28396.1 | 5792109 |
| 2 | DU502334_443.1 | 19287623 |
| 2 | OAR2_21543423.1 | 21543423 |
| 2 | s50613.1 | 46847073 |
| 2 | s72174.1 | 70839178 |
| 2 | OAR2_100345751.1 | 100345751 |
| 2 | OAR2_132019304.1 | 132019304 |
| 2 | OAR2_142060351.1 | 142060351 |
| 2 | OAR2_156250273.1 | 156250273 |
| 2 | OAR2_157237046.1 | 157237046 |
| 2 | OAR2_165104317.1 | 165104317 |
| 2 | OAR2_209746295.1 | 209746295 |
| 2 | OAR2_211051742.1 | 211051742 |
| 2 | OAR2_217238961.1 | 217238961 |
| 2 | s55106.1 | 228216883 |
| 2 | OAR2_229781196.1 | 229781196 |
| 2 | s67306.1 | 253034708 |
| 2 | OAR2_257849665.1 | 257849665 |
| 2 | s43169.1 | 261504511 |
| 3 | OAR3_2803962.1 | 2803962 |
| 3 | s34275.1 | 6719786 |
| 3 | OAR3_16051606.1 | 16051606 |
| 3 | s57632.1 | 22674669 |
| 3 | OAR3_25248302.1 | 25248302 |
| 3 | OAR3_31172991.1 | 31172991 |
| 3 | s14975.1 | 44671476 |
| 3 | s48852.1 | 60323836 |
| 3 | OAR3_76177373.1 | 76177373 |
| 3 | OAR3_80099118.1 | 80099118 |
| 3 | s18744.1 | 126614957 |
| 3 | OAR3_127402873.1 | 127402873 |
| 3 | OAR3_151315027.1 | 151315027 |
| 3 | s66461.1 | 167783642 |
| 3 | OAR3_181981901.1 | 181981901 |
| 3 | OAR3_199355985.1 | 199355985 |
| 3 | OAR3_205542104.1 | 205542104 |
| 3 | s48060.1 | 209529081 |
| 3 | OAR3_211514604.1 | 211514604 |
| 3 | OAR3_218986335.1 | 218986335 |
| 3 | s24635.1 | 221763355 |
| 3 | s26496.1 | 230533654 |
| 3 | OAR3_233232424.1 | 233232424 |
| 3 | s22560.1 | 237632580 |
| 4 | OAR4_1156731.1 | 1156731 |
| 4 | s31124.1 | 5309191 |
| 4 | OAR4_9646078.1 | 9646078 |
| 4 | OAR4_12843394.1 | 12843394 |
| 4 | OAR4_48471516.1 | 48471516 |
| 4 | OAR4_50013760.1 | 50013760 |
| 4 | OAR4_58493210.1 | 58493210 |
| 4 | OAR4_67200551.1 | 67200551 |
| 4 | OAR4_76592305.1 | 76592305 |
| 4 | OAR4_76692624.1 | 76692624 |
| 4 | OAR4_107537251.1 | 107537251 |
| 4 | OAR4_110428220.1 | 110428220 |
| 4 | DU176899_379.1 | 117231908 |
| 4 | OAR4_123683864.1 | 123683864 |
| 4 | OAR4_124514934.1 | 124514934 |
| 4 | OAR4_126907103.1 | 126907103 |
| 5 | s01567.1 | 2759117 |
| 5 | OAR5_6445442.1 | 6445442 |
| 5 | s30649.1 | 11767765 |
| 5 | s18792.1 | 16439394 |
| 5 | s07825.1 | 47964597 |
| 5 | s19690.1 | 74296498 |
| 5 | OAR5_91733625.1 | 91733625 |
| 5 | s50174.1 | 98289570 |
| 5 | OAR5_101792466.1 | 101792466 |
| 5 | OAR5_104273659.1 | 104273659 |
| 5 | OAR5_111467301.1 | 111467301 |
| 5 | OAR5_111922624.1 | 111922624 |
| 6 | OAR6_4713180.1 | 4713180 |
| 6 | OAR6_6431454.1 | 6431454 |
| 6 | OAR6_16572510.1 | 16572510 |
| 6 | OAR6_20938615.1 | 20938615 |
| 6 | OAR6_24042528.1 | 24042528 |
| 6 | OAR6_34609120.1 | 34609120 |
| 6 | OAR6_35314271.1 | 35314271 |
| 6 | DU194639_560.1 | 62375527 |
| 6 | s05120.1 | 65973084 |
| 6 | s64898.1 | 74458739 |
| 6 | OAR6_102195983.1 | 102195983 |
| 6 | OAR6_107118527.1 | 107118527 |
| 6 | s58059.1 | 125943177 |
| 6 | OAR6_127794408_X.1 | 127794409 |
| 6 | s55582.1 | 128936598 |
| 7 | OAR7_1317777.1 | 1317777 |
| 7 | OAR7_5781390_X.1 | 5781391 |
| 7 | s03550.1 | 25402804 |
| 7 | OAR7_27944184.1 | 27944184 |
| 7 | s11708.1 | 38467050 |
| 7 | s49523.1 | 38943243 |
| 7 | s68841.1 | 81773651 |
| 7 | s47322.1 | 88628319 |
| 7 | s33311.1 | 90453576 |
| 7 | s58752.1 | 105547551 |
| 7 | OAR7_108058672.1 | 108058672 |
| 8 | OAR8_181325.1 | 181325 |
| 8 | OAR8_6595131.1 | 6595131 |
| 8 | s60361.1 | 25145108 |
| 8 | OAR8_32777282.1 | 32777282 |
| 8 | OAR8_55933152.1 | 55933152 |
| 8 | OAR8_64933647.1 | 64933647 |
| 8 | OAR8_76529883.1 | 76529883 |
| 8 | OAR8_81191251.1 | 81191251 |
| 8 | s41250.1 | 88621901 |
| 8 | s42859.1 | 92383226 |
| 8 | OAR8_95443730.1 | 95443730 |
| 9 | s03522.1 | 482053 |
| 9 | OAR9_3787356.1 | 3787356 |
| 9 | s14559.1 | 17601739 |
| 9 | s10902.1 | 37474056 |
| 9 | s33596.1 | 41396241 |
| 9 | s54630.1 | 43586314 |
| 9 | OAR9_62169543.1 | 62169543 |
| 9 | OAR9_76409786.1 | 76409786 |
| 9 | OAR9_84837431.1 | 84837431 |
| 9 | OAR9_89681314.1 | 89681314 |
| 9 | OAR9_93946383.1 | 93946383 |
| 9 | s03736.1 | 100141994 |
| 10 | OAR10_617510.1 | 617510 |
| 10 | OAR10_1797787.1 | 1797787 |
| 10 | OAR10_8467750.1 | 8467750 |
| 10 | s57500.1 | 30446995 |
| 10 | s39805.1 | 33115863 |
| 10 | OAR10_52323600.1 | 52323600 |
| 10 | OAR10_56572554.1 | 56572554 |
| 10 | s15088.1 | 69146077 |
| 10 | OAR10_72786672.1 | 72786672 |
| 10 | OAR10_79823099.1 | 79823099 |
| 10 | OAR10_86679587.1 | 86679587 |
| 10 | OAR10_91011990.1 | 91011990 |
| 10 | s46635.1 | 93055148 |
| 11 | OAR11_2909058.1 | 2909058 |
| 11 | OAR11_5031247.1 | 5031247 |
| 11 | s18615.1 | 10888235 |
| 11 | s26369.1 | 15988986 |
| 11 | OAR11_33427461.1 | 33427461 |
| 11 | s45109.1 | 37299458 |
| 11 | OAR11_43769313.1 | 43769313 |
| 11 | s66645.1 | 53609274 |
| 11 | s15677.1 | 57091667 |
| 11 | OAR11_62965533.1 | 62965533 |
| 11 | s37124.1 | 65978069 |
| 12 | s09930.1 | 4059636 |
| 12 | OAR12_9576010.1 | 9576010 |
| 12 | OAR12_20662545.1 | 20662545 |
| 12 | OAR12_24096729.1 | 24096729 |
| 12 | OAR12_32481363.1 | 32481363 |
| 12 | OAR12_51213999.1 | 51213999 |
| 12 | s05958.1 | 52699410 |
| 12 | s72450.1 | 55415907 |
| 12 | OAR12_68075884.1 | 68075884 |
| 12 | OAR12_71458988.1 | 71458988 |
| 12 | OAR12_84863239.1 | 84863239 |
| 12 | OAR12_84938649.1 | 84938649 |
| 13 | OAR13_201555.1 | 201555 |
| 13 | OAR13_6156528.1 | 6156528 |
| 13 | OAR13_12683522.1 | 12683522 |
| 13 | OAR13_16254632.1 | 16254632 |
| 13 | s04798.1 | 19488357 |
| 13 | OAR13_35098031.1 | 35098031 |
| 13 | s58872.1 | 41559144 |
| 13 | OAR13_56551494.1 | 56551494 |
| 13 | s54263.1 | 74121874 |
| 13 | s38696.1 | 80047544 |
| 13 | s31649.1 | 84208796 |
| 14 | s11472.1 | 668805 |
| 14 | OAR14_4168859.1 | 4168859 |
| 14 | s32850.1 | 7122285 |
| 14 | OAR14_21572610_X.1 | 21572611 |
| 14 | s21110.1 | 30514165 |
| 14 | s18349.1 | 37689386 |
| 14 | s20490.1 | 43056636 |
| 14 | OAR14_57012757.1 | 57012757 |
| 14 | OAR14_65487938.1 | 65487938 |
| 14 | OAR14_67405636.1 | 67405636 |
| 15 | OAR15_1968953.1 | 1968953 |
| 15 | s15392.1 | 14602743 |
| 15 | OAR15_21189336.1 | 21189336 |
| 15 | s71542.1 | 27611384 |
| 15 | s72653.1 | 33253268 |
| 15 | OAR15_39821855_X.1 | 39821856 |
| 15 | OAR15_43509128.1 | 43509128 |
| 15 | OAR15_57530994.1 | 57530994 |
| 15 | s46520.1 | 64565148 |
| 15 | OAR15_71392435_X.1 | 71392436 |
| 15 | OAR15_75169402.1 | 75169402 |
| 15 | OAR15_77894279.1 | 77894279 |
| 15 | s32555.1 | 87597074 |
| 15 | OAR15_89389345.1 | 89389345 |
| 16 | s45546.1 | 2296445 |
| 16 | s61872.1 | 22097589 |
| 16 | OAR16_24475720.1 | 24475720 |
| 16 | OAR16_26775858.1 | 26775858 |
| 16 | OAR16_37519511.1 | 37519511 |
| 16 | OAR16_50704170.1 | 50704170 |
| 16 | OAR16_57881990.1 | 57881990 |
| 16 | OAR16_66139716.1 | 66139716 |
| 16 | s17233.1 | 72947468 |
| 16 | s08594.1 | 73584849 |
| 17 | s27585.1 | 2113004 |
| 17 | OAR17_16852424.1 | 16852424 |
| 17 | OAR17_18141691.1 | 18141691 |
| 17 | OAR17_26733934.1 | 26733934 |
| 17 | s56085.1 | 45965274 |
| 17 | s66447.1 | 47624166 |
| 17 | OAR17_66088115.1 | 66088115 |
| 17 | s66157.1 | 77249179 |
| 17 | OAR17_77496917.1 | 77496917 |
| 18 | OAR18_5630311.1 | 5630311 |
| 18 | s59944.1 | 11832022 |
| 18 | s68189.1 | 16688642 |
| 18 | s64801.1 | 22232534 |
| 18 | s69804.1 | 27168228 |
| 18 | OAR18_36079788.1 | 36079788 |
| 18 | OAR18_51687907.1 | 51687907 |
| 18 | OAR18_59513052.1 | 59513052 |
| 18 | s75539.1 | 66828017 |
| 18 | s67734.1 | 68738392 |
| 18 | s39671.1 | 71433226 |
| 19 | OAR19_2917094.1 | 2917094 |
| 19 | OAR19_10439681.1 | 10439681 |
| 19 | s63459.1 | 12438098 |
| 19 | s31311.1 | 16981764 |
| 19 | OAR19_44043850_X.1 | 44043851 |
| 19 | OAR19_60258539.1 | 60258539 |
| 19 | DU411432_523.1 | 60468232 |
| 19 | OAR19_64803054.1 | 64803054 |
| 20 | s59474.1 | 2035597 |
| 20 | OAR20_10199126.1 | 10199126 |
| 20 | OAR20_13093640.1 | 13093640 |
| 20 | s34524.1 | 26089937 |
| 20 | DU210150_274.1 | 26218506 |
| 20 | s12116.1 | 44695478 |
| 20 | OAR20_51469250.1 | 51469250 |
| 20 | s64295.1 | 54136727 |
| 21 | s55849.1 | 495784 |
| 21 | OAR21_7464532.1 | 7464532 |
| 21 | s09740.1 | 18723983 |
| 21 | OAR21_24084777.1 | 24084777 |
| 21 | OAR21_31010355.1 | 31010355 |
| 21 | s43298.1 | 34276716 |
| 21 | s55490.1 | 43754091 |
| 21 | s52755.1 | 50505230 |
| 21 | s69566.1 | 52715266 |
| 22 | OAR22_3936789.1 | 3936789 |
| 22 | s18005.1 | 6725139 |
| 22 | OAR22_11412310.1 | 11412310 |
| 22 | OAR22_23763191.1 | 23763191 |
| 22 | s66275.1 | 37757233 |
| 22 | s11624.1 | 40073316 |
| 22 | OAR22_40609932.1 | 40609932 |
| 22 | s31685.1 | 48529765 |
| 22 | s40969.1 | 53963300 |
| 23 | OAR23_429075.1 | 429075 |
| 23 | OAR23_4607326.1 | 4607326 |
| 23 | OAR23_12072551.1 | 12072551 |
| 23 | s41429.1 | 47283990 |
| 23 | OAR23_51500282.1 | 51500282 |
| 23 | OAR23_60936698.1 | 60936698 |
| 23 | OAR23_63493732.1 | 63493732 |
| 24 | s44109.1 | 5212064 |
| 24 | s54640.1 | 6999044 |
| 24 | s12995.1 | 8824327 |
| 24 | OAR24_15159567.1 | 15159567 |
| 24 | s07059.1 | 18711283 |
| 24 | OAR24_33013180.1 | 33013180 |
| 24 | s14581.1 | 44407756 |
| 25 | s33329.1 | 245910 |
| 25 | s54695.1 | 612009 |
| 25 | OAR25_9819368.1 | 9819368 |
| 25 | s02748.1 | 11783910 |
| 25 | s40313.1 | 27545426 |
| 25 | OAR25_31570574.1 | 31570574 |
| 25 | s04816.1 | 36325382 |
| 25 | s62494.1 | 38693343 |
| 25 | s69770.1 | 41817988 |
| 25 | s54764.1 | 45843550 |
| 26 | s10134.1 | 439317 |
| 26 | s21455.1 | 3647454 |
| 26 | s64852.1 | 16440017 |
| 26 | OAR26_37810731_X.1 | 37810732 |
| 26 | OAR26_45654578.1 | 45654578 |
| 26 | s68179.1 | 48432560 |

**Table S2 :** Summary statistics of pruned pedigrees for all univariate models 1. Pruned pedigrees only include individuals which are either phenotyped, or related to two or more phenotyped individuals. All pedigree statistics were obtained using the R package PEDANTICS ([Morrissey & Wilson 2010](#_ENREF_36)).

| **A) Neonates and lambs** |  |  |  |  |  |  |
| --- | --- | --- | --- | --- | --- | --- |
| **Pedigree 1** | **Birthweight** | **Foreleg** | **Hindleg** | **Weight** | **Metacarpal length** | **Jaw length** |
| records | 3642 | 2067 | 2131 | 2234 | 1879 | 2063 |
| maternities | 3246 | 1804 | 1866 | 1946 | 1626 | 1779 |
| paternities | 1672 | 1095 | 1132 | 1157 | 1025 | 1078 |
| full sibs | 114 | 68 | 68 | 72 | 44 | 45 |
| maternal sibs | 8833 | 3335 | 3610 | 3783 | 2753 | 3127 |
| maternal half sibs | 8719 | 3267 | 3542 | 3711 | 2709 | 3082 |
| paternal sibs | 9111 | 4040 | 4293 | 4462 | 3509 | 3838 |
| paternal half sibs | 8997 | 3972 | 4225 | 4390 | 3465 | 3793 |
| maternal grandmothers | 2338 | 1346 | 1387 | 1430 | 1214 | 1284 |
| maternal grandfathers | 1374 | 780 | 804 | 815 | 740 | 765 |
| paternal grandmothers | 888 | 593 | 615 | 626 | 557 | 571 |
| paternal grandfathers | 689 | 477 | 490 | 491 | 424 | 426 |
| maximum pedigree depth | 9 | 9 | 9 | 9 | 8 | 8 |
| founders | 335 | 199 | 201 | 224 | 196 | 222 |
| mean maternal sibsip size | 4.386486486 | 3.221428571 | 3.320284698 | 3.326495726 | 3.056390977 | 3.104712042 |
| mean paternal sibsip size | 4.435013263 | 3.724489796 | 3.773333333 | 3.818481848 | 3.660714286 | 3.717241379 |
| non-zero F | 93 | 62 | 62 | 62 | 53 | 54 |
| F > 0.125 | 7 | 2 | 2 | 2 | 2 | 2 |
| mean pairwise relatedness | 0.003666534 | 0.005055297 | 0.00504176 | 0.004749864 | 0.00518543 | 0.00470966 |
| pairwise relatedness>=0.125 | 0.011621111 | 0.016040569 | 0.016033822 | 0.015158799 | 0.016627928 | 0.015191215 |
| pairwise relatedness>=0.25 | 0.004284296 | 0.006349255 | 0.006301787 | 0.005947253 | 0.006766112 | 0.006102627 |
| pairwise relatedness>=0.5 | 0.000760604 | 0.001393773 | 0.001354915 | 0.00127653 | 0.001531982 | 0.001368154 |
|  |  |  |  |  |  |  |
| **Pedigree 2** | **Birthweight** | **Foreleg** | **Hindleg** | **Weight** | **Metacarpal length** | **Jaw length** |
| records | 4238 | 2518 | 2581 | 2670 | 2432 | 2612 |
| maternities | 3818 | 2214 | 2275 | 2348 | 2133 | 2285 |
| paternities | 2960 | 1885 | 1936 | 1955 | 1855 | 1922 |
| full sibs | 238 | 124 | 129 | 133 | 86 | 82 |
| maternal sibs | 10621 | 4141 | 4428 | 4627 | 3863 | 4288 |
| maternal half sibs | 10383 | 4017 | 4299 | 4494 | 3777 | 4206 |
| paternal sibs | 22533 | 9332 | 9773 | 9928 | 9165 | 9601 |
| paternal half sibs | 22295 | 9208 | 9644 | 9795 | 9079 | 9519 |
| maternal grandmothers | 3114 | 1836 | 1884 | 1935 | 1782 | 1873 |
| maternal grandfathers | 2464 | 1528 | 1569 | 1573 | 1489 | 1522 |
| paternal grandmothers | 1736 | 1074 | 1111 | 1126 | 1072 | 1101 |
| paternal grandfathers | 1716 | 1116 | 1133 | 1130 | 1117 | 1140 |
| maximum pedigree depth | 10 | 9 | 9 | 9 | 9 | 9 |
| founders | 289 | 191 | 192 | 212 | 185 | 210 |
| mean maternal sibsip size | 4.408775982 | 3.255882353 | 3.340675477 | 3.373563218 | 3.174107143 | 3.241134752 |
| mean paternal sibsip size | 5.352622061 | 4.170353982 | 4.227074236 | 4.268558952 | 4.206349206 | 4.242825607 |
| non-zero F | 472 | 295 | 296 | 296 | 275 | 280 |
| F > 0.125 | 16 | 10 | 10 | 10 | 6 | 7 |
| mean pairwise relatedness | 0.006640839 | 0.008021396 | 0.00797677 | 0.007609781 | 0.008124415 | 0.007503982 |
| pairwise relatedness>=0.125 | 0.017433221 | 0.021491664 | 0.021403278 | 0.020468326 | 0.022299343 | 0.020614868 |
| pairwise relatedness>=0.25 | 0.005549106 | 0.007403508 | 0.007348873 | 0.007017736 | 0.007725054 | 0.007087754 |
| pairwise relatedness>=0.5 | 0.000784344 | 0.001338318 | 0.001308909 | 0.00125003 | 0.001384258 | 0.001263356 |
|  |  |  |  |  |  |  |
| **B) Yearlings** |  |  |  |  |  |  |
| **Pedigree 1** |  | **Foreleg** | **Hindleg** | **Weight** | **Metacarpal length** | **Jaw length** |
| records |  | 1066 | 1078 | 1088 | 503 | 594 |
| maternities |  | 870 | 880 | 888 | 375 | 447 |
| paternities |  | 529 | 539 | 538 | 213 | 236 |
| full sibs |  | 13 | 13 | 13 | 6 | 8 |
| maternal sibs |  | 885 | 906 | 916 | 222 | 290 |
| maternal half sibs |  | 872 | 893 | 903 | 216 | 282 |
| paternal sibs |  | 1148 | 1196 | 1190 | 303 | 397 |
| paternal half sibs |  | 1135 | 1183 | 1177 | 297 | 389 |
| maternal grandmothers |  | 602 | 609 | 611 | 236 | 277 |
| maternal grandfathers |  | 369 | 371 | 370 | 138 | 148 |
| paternal grandmothers |  | 302 | 309 | 309 | 98 | 105 |
| paternal grandfathers |  | 235 | 239 | 239 | 78 | 79 |
| maximum pedigree depth |  | 8 | 8 | 8 | 7 | 7 |
| founders |  | 138 | 139 | 144 | 80 | 103 |
| mean maternal sibsip size |  | 2.236503856 | 2.250639386 | 2.248101266 | 1.712328767 | 1.752941176 |
| mean paternal sibsip size |  | 2.890710383 | 2.929347826 | 2.923913043 | 2.340659341 | 2.510638298 |
| non-zero F |  | 24 | 25 | 25 | 3 | 3 |
| F > 0.125 |  | 0 | 0 | 0 | 0 | 0 |
| mean pairwise relatedness |  | 0.00599653 | 0.006016722 | 0.005907931 | 0.007336855 | 0.0062511 |
| pairwise relatedness>=0.125 |  | 0.01973945 | 0.019837968 | 0.019464663 | 0.025401377 | 0.021865649 |
| pairwise relatedness>=0.25 |  | 0.008762519 | 0.008759645 | 0.008617891 | 0.013219488 | 0.011264983 |
| pairwise relatedness>=0.5 |  | 0.002489232 | 0.002470271 | 0.002436888 | 0.004704839 | 0.003923439 |
|  |  |  |  |  |  |  |
| **Pedigree 2** |  | **Foreleg** | **Hindleg** | **Weight** | **Metacarpal length** | **Jaw length** |
| records |  | 1258 | 1271 | 1279 | 700 | 789 |
| maternities |  | 1045 | 1057 | 1063 | 534 | 614 |
| paternities |  | 867 | 879 | 878 | 444 | 468 |
| full sibs |  | 25 | 25 | 25 | 8 | 10 |
| maternal sibs |  | 1100 | 1129 | 1135 | 353 | 438 |
| maternal half sibs |  | 1075 | 1104 | 1110 | 345 | 428 |
| paternal sibs |  | 2319 | 2386 | 2385 | 722 | 844 |
| paternal half sibs |  | 2294 | 2361 | 2360 | 714 | 834 |
| maternal grandmothers |  | 797 | 806 | 810 | 384 | 430 |
| maternal grandfathers |  | 645 | 649 | 648 | 292 | 298 |
| paternal grandmothers |  | 513 | 522 | 522 | 227 | 243 |
| paternal grandfathers |  | 450 | 455 | 455 | 180 | 183 |
| maximum pedigree depth |  | 10 | 10 | 10 | 9 | 9 |
| founders |  | 140 | 141 | 145 | 106 | 124 |
| mean maternal sibsip size |  | 2.26681128 | 2.282937365 | 2.28111588 | 1.791946309 | 1.832835821 |
| mean paternal sibsip size |  | 3.141304348 | 3.173285199 | 3.16967509 | 2.466666667 | 2.629213483 |
| non-zero F |  | 106 | 107 | 107 | 31 | 32 |
| F > 0.125 |  | 1 | 1 | 1 | 1 | 1 |
| mean pairwise relatedness |  | 0.008269936 | 0.008294077 | 0.008202962 | 0.008484468 | 0.007521488 |
| pairwise relatedness>=0.125 |  | 0.024573359 | 0.024683893 | 0.02440042 | 0.027488249 | 0.024508952 |
| pairwise relatedness>=0.25 |  | 0.009877911 | 0.009863893 | 0.009756742 | 0.012879624 | 0.011377893 |
| pairwise relatedness>=0.5 |  | 0.002453668 | 0.002434688 | 0.002410432 | 0.004038422 | 0.003519201 |
|  |  |  |  |  |  |  |
| **C) Adults** |  |  |  |  |  |  |
| **Pedigree 1** |  | **Foreleg** | **Hindleg** | **Weight** | **Metacarpal length** | **Jaw length** |
| records |  | 987 | 998 | 995 | 847 | 888 |
| maternities |  | 801 | 812 | 809 | 671 | 703 |
| paternities |  | 482 | 487 | 484 | 408 | 416 |
| full sibs |  | 7 | 7 | 7 | 6 | 6 |
| maternal sibs |  | 740 | 761 | 759 | 542 | 597 |
| maternal half sibs |  | 733 | 754 | 752 | 536 | 591 |
| paternal sibs |  | 882 | 901 | 898 | 658 | 684 |
| paternal half sibs |  | 875 | 894 | 891 | 652 | 678 |
| maternal grandmothers |  | 551 | 556 | 553 | 463 | 469 |
| maternal grandfathers |  | 328 | 330 | 327 | 268 | 272 |
| paternal grandmothers |  | 265 | 269 | 266 | 219 | 225 |
| paternal grandfathers |  | 214 | 216 | 214 | 184 | 188 |
| maximum pedigree depth |  | 9 | 9 | 9 | 8 | 8 |
| founders |  | 136 | 136 | 136 | 122 | 135 |
| mean maternal sibsip size |  | 2.113456464 | 2.12565445 | 2.128947368 | 1.979351032 | 2.031791908 |
| mean paternal sibsip size |  | 2.677777778 | 2.690607735 | 2.703910615 | 2.457831325 | 2.491017964 |
| non-zero F |  | 26 | 26 | 26 | 20 | 21 |
| F > 0.125 |  | 2 | 2 | 2 | 2 | 2 |
| mean pairwise relatedness |  | 0.005833749 | 0.005805895 | 0.005788197 | 0.006011762 | 0.005733277 |
| pairwise relatedness>=0.125 |  | 0.018931711 | 0.018862198 | 0.018836638 | 0.019883834 | 0.01900322 |
| pairwise relatedness>=0.25 |  | 0.008797943 | 0.008745676 | 0.008754032 | 0.009553953 | 0.009072488 |
| pairwise relatedness>=0.5 |  | 0.002655207 | 0.00262913 | 0.002632883 | 0.00303114 | 0.002861655 |
|  |  |  |  |  |  |  |
| **Pedigree 2** |  | **Foreleg** | **Hindleg** | **Weight** | **Metacarpal length** | **Jaw length** |
| records |  | 1143 | 1151 | 1149 | 979 | 1024 |
| maternities |  | 933 | 941 | 939 | 781 | 821 |
| paternities |  | 781 | 785 | 782 | 646 | 656 |
| full sibs |  | 14 | 14 | 14 | 10 | 10 |
| maternal sibs |  | 909 | 924 | 924 | 687 | 755 |
| maternal half sibs |  | 895 | 910 | 910 | 677 | 745 |
| paternal sibs |  | 1819 | 1829 | 1824 | 1201 | 1233 |
| paternal half sibs |  | 1805 | 1815 | 1810 | 1191 | 1223 |
| maternal grandmothers |  | 701 | 705 | 703 | 574 | 585 |
| maternal grandfathers |  | 563 | 566 | 563 | 438 | 441 |
| paternal grandmothers |  | 439 | 443 | 440 | 347 | 360 |
| paternal grandfathers |  | 427 | 427 | 424 | 326 | 330 |
| maximum pedigree depth |  | 9 | 9 | 9 | 9 | 9 |
| founders |  | 133 | 133 | 133 | 123 | 133 |
| mean maternal sibsip size |  | 2.179906542 | 2.188372093 | 2.193925234 | 2.055263158 | 2.099744246 |
| mean paternal sibsip size |  | 2.819494585 | 2.823741007 | 2.833333333 | 2.553359684 | 2.5625 |
| non-zero F |  | 86 | 86 | 86 | 49 | 50 |
| F > 0.125 |  | 2 | 2 | 2 | 2 | 2 |
| mean pairwise relatedness |  | 0.008023253 | 0.007980622 | 0.007957268 | 0.007494083 | 0.007121222 |
| pairwise relatedness>=0.125 |  | 0.024132272 | 0.024003324 | 0.023977826 | 0.02351216 | 0.022389342 |
| pairwise relatedness>=0.25 |  | 0.010135554 | 0.010069127 | 0.010072385 | 0.0104965 | 0.009962274 |
| pairwise relatedness>=0.5 |  | 0.002653784 | 0.002635138 | 0.002636742 | 0.003007952 | 0.002846637 |

**Table S3:** Summary statistics of pruned pedigrees for maternal genetic effect in all univariate models 2. Pruned pedigrees only include mothers of phenotyped individuals, or individuals who are related to two or more mothers of phenotyped individuals. All pedigree statistics were obtained using the R package PEDANTICS ([Morrissey & Wilson 2010](#_ENREF_36)).

| **A) Neonates and lambs** |  |  |  |  |  |  |
| --- | --- | --- | --- | --- | --- | --- |
| **Pedigree 1** | **Birthweight** | **Foreleg** | **Hindleg** | **Weight** | **Metacarpal length** | **Jaw length** |
| records | 772 | 632 | 634 | 641 | 607 | 631 |
| maternities | 603 | 487 | 489 | 495 | 461 | 480 |
| paternities | 346 | 285 | 286 | 286 | 279 | 282 |
| full sibs | 4 | 3 | 3 | 3 | 3 | 3 |
| maternal sibs | 493 | 368 | 370 | 376 | 336 | 353 |
| maternal half sibs | 489 | 365 | 367 | 373 | 333 | 350 |
| paternal sibs | 557 | 373 | 379 | 379 | 409 | 416 |
| paternal half sibs | 553 | 370 | 376 | 376 | 406 | 413 |
| maternal grandmothers | 373 | 302 | 302 | 303 | 289 | 296 |
| maternal grandfathers | 223 | 191 | 193 | 193 | 178 | 178 |
| paternal grandmothers | 181 | 150 | 150 | 150 | 140 | 140 |
| paternal grandfathers | 148 | 120 | 120 | 120 | 120 | 120 |
| maximum pedigree depth | 7 | 7 | 7 | 7 | 7 | 7 |
| founders | 122 | 99 | 99 | 101 | 100 | 105 |
| mean maternal sibsip size | 2.01 | 1.894941634 | 1.902723735 | 1.903846154 | 1.87398374 | 1.882352941 |
| mean paternal sibsip size | 2.621212121 | 2.415254237 | 2.423728814 | 2.423728814 | 2.405172414 | 2.431034483 |
| non-zero F | 10 | 8 | 8 | 8 | 8 | 8 |
| F > 0.125 | 0 | 0 | 0 | 0 | 0 | 0 |
| mean pairwise relatedness | 0.005961993 | 0.006785743 | 0.006781338 | 0.006665001 | 0.006969297 | 0.006613463 |
| pairwise relatedness>=0.125 | 0.020130643 | 0.022828943 | 0.022824565 | 0.022430772 | 0.02374389 | 0.022574397 |
| pairwise relatedness>=0.25 | 0.009862032 | 0.011464623 | 0.011462118 | 0.011276326 | 0.012086711 | 0.011455739 |
| pairwise relatedness>=0.5 | 0.003205581 | 0.003891753 | 0.003882169 | 0.003827028 | 0.004045215 | 0.003853797 |
|  |  |  |  |  |  |  |
| **Pedigree 2** | **Birthweight** | **Foreleg** | **Hindleg** | **Weight** | **Metacarpal length** | **Jaw length** |
| records | 1012 | 847 | 849 | 854 | 854 | 875 |
| maternities | 806 | 669 | 671 | 676 | 674 | 692 |
| paternities | 660 | 554 | 555 | 556 | 563 | 566 |
| full sibs | 9 | 7 | 7 | 7 | 5 | 5 |
| maternal sibs | 694 | 534 | 535 | 539 | 535 | 557 |
| maternal half sibs | 685 | 527 | 528 | 532 | 530 | 552 |
| paternal sibs | 1264 | 957 | 963 | 964 | 1001 | 1006 |
| paternal half sibs | 1255 | 950 | 956 | 957 | 996 | 1001 |
| maternal grandmothers | 586 | 480 | 481 | 483 | 495 | 505 |
| maternal grandfathers | 466 | 392 | 394 | 394 | 403 | 403 |
| paternal grandmothers | 372 | 307 | 307 | 308 | 307 | 310 |
| paternal grandfathers | 346 | 282 | 282 | 282 | 289 | 289 |
| maximum pedigree depth | 9 | 8 | 8 | 8 | 8 | 8 |
| founders | 134 | 111 | 111 | 113 | 113 | 117 |
| mean maternal sibsip size | 2.050890585 | 1.944767442 | 1.950581395 | 1.948126801 | 1.942363112 | 1.95480226 |
| mean paternal sibsip size | 2.693877551 | 2.552995392 | 2.557603687 | 2.562211982 | 2.502222222 | 2.515555556 |
| non-zero F | 66 | 47 | 47 | 47 | 52 | 52 |
| F > 0.125 | 1 | 1 | 1 | 1 | 1 | 1 |
| mean pairwise relatedness | 0.007695635 | 0.008360477 | 0.008344167 | 0.008272291 | 0.008379023 | 0.008112978 |
| pairwise relatedness>=0.125 | 0.023613766 | 0.026066132 | 0.026023957 | 0.025799561 | 0.026159223 | 0.025339 |
| pairwise relatedness>=0.25 | 0.010207872 | 0.01171706 | 0.01170078 | 0.011602527 | 0.01177824 | 0.011381497 |
| pairwise relatedness>=0.5 | 0.002889168 | 0.003441433 | 0.003433562 | 0.003409924 | 0.00341816 | 0.003310886 |
|  |  |  |  |  |  |  |
| **B) Yearlings** |  |  |  |  |  |  |
| **Pedigree 1** |  | **Foreleg** | **Hindleg** | **Weight** | **Metacarpal length** | **Jaw length** |
| records |  | 446 | 450 | 450 | 244 | 258 |
| maternities |  | 329 | 333 | 333 | 164 | 175 |
| paternities |  | 205 | 205 | 205 | 92 | 95 |
| full sibs |  | 2 | 2 | 2 | 2 | 2 |
| maternal sibs |  | 185 | 191 | 191 | 83 | 90 |
| maternal half sibs |  | 183 | 189 | 189 | 81 | 88 |
| paternal sibs |  | 252 | 252 | 252 | 91 | 105 |
| paternal half sibs |  | 250 | 250 | 250 | 89 | 103 |
| maternal grandmothers |  | 196 | 198 | 198 | 78 | 82 |
| maternal grandfathers |  | 120 | 121 | 121 | 51 | 55 |
| paternal grandmothers |  | 111 | 111 | 111 | 38 | 39 |
| paternal grandfathers |  | 85 | 85 | 85 | 28 | 28 |
| maximum pedigree depth |  | 7 | 7 | 7 | 6 | 6 |
| founders |  | 76 | 76 | 76 | 50 | 53 |
| mean maternal sibsip size |  | 1.661616162 | 1.673366834 | 1.673366834 | 1.607843137 | 1.62037037 |
| mean paternal sibsip size |  | 2.277777778 | 2.277777778 | 2.277777778 | 2.090909091 | 2.159090909 |
| non-zero F |  | 4 | 4 | 4 | 0 | 0 |
| F > 0.125 |  | 0 | 0 | 0 | 0 | 0 |
| mean pairwise relatedness |  | 0.008204515 | 0.008136352 | 0.008136352 | 0.010060379 | 0.009655235 |
| pairwise relatedness>=0.125 |  | 0.028084849 | 0.027834694 | 0.027834694 | 0.034507185 | 0.033300154 |
| pairwise relatedness>=0.25 |  | 0.014974555 | 0.014837911 | 0.014837911 | 0.021149565 | 0.020239496 |
| pairwise relatedness>=0.5 |  | 0.00540132 | 0.005345212 | 0.005345212 | 0.008702692 | 0.008204386 |
|  |  |  |  |  |  |  |
| **Pedigree 2** |  | **Foreleg** | **Hindleg** | **Weight** | **Metacarpal length** | **Jaw length** |
| records |  | 563 | 566 | 566 | 346 | 362 |
| maternities |  | 428 | 431 | 431 | 243 | 255 |
| paternities |  | 343 | 343 | 343 | 182 | 189 |
| full sibs |  | 4 | 4 | 4 | 2 | 2 |
| maternal sibs |  | 270 | 275 | 275 | 132 | 146 |
| maternal half sibs |  | 266 | 271 | 271 | 130 | 144 |
| paternal sibs |  | 479 | 479 | 479 | 183 | 198 |
| paternal half sibs |  | 475 | 475 | 475 | 181 | 196 |
| maternal grandmothers |  | 276 | 277 | 277 | 144 | 147 |
| maternal grandfathers |  | 229 | 230 | 230 | 113 | 118 |
| paternal grandmothers |  | 195 | 195 | 195 | 87 | 89 |
| paternal grandfathers |  | 169 | 169 | 169 | 61 | 62 |
| maximum pedigree depth |  | 9 | 9 | 9 | 6 | 6 |
| founders |  | 86 | 86 | 86 | 61 | 64 |
| mean maternal sibsip size |  | 1.725806452 | 1.737903226 | 1.737903226 | 1.609271523 | 1.634615385 |
| mean paternal sibsip size |  | 2.333333333 | 2.333333333 | 2.333333333 | 2.11627907 | 2.123595506 |
| non-zero F |  | 20 | 20 | 20 | 8 | 8 |
| F > 0.125 |  | 1 | 1 | 1 | 1 | 1 |
| mean pairwise relatedness |  | 0.009420017 | 0.009381469 | 0.009381469 | 0.010319176 | 0.009975743 |
| pairwise relatedness>=0.125 |  | 0.030878049 | 0.030732668 | 0.030732668 | 0.035251738 | 0.034067431 |
| pairwise relatedness>=0.25 |  | 0.015138777 | 0.015041121 | 0.015041121 | 0.019200804 | 0.018441713 |
| pairwise relatedness>=0.5 |  | 0.004911411 | 0.004878201 | 0.004878201 | 0.007170981 | 0.006841034 |
|  |  |  |  |  |  |  |
| **C) Adults** |  |  |  |  |  |  |
| **Pedigree 1** |  | **Foreleg** | **Hindleg** | **Weight** | **Metacarpal length** | **Jaw length** |
| records |  | 441 | 444 | 442 | 348 | 348 |
| maternities |  | 330 | 332 | 330 | 247 | 247 |
| paternities |  | 194 | 196 | 195 | 147 | 147 |
| full sibs |  | 2 | 2 | 2 | 2 | 2 |
| maternal sibs |  | 184 | 186 | 184 | 134 | 134 |
| maternal half sibs |  | 182 | 184 | 182 | 132 | 132 |
| paternal sibs |  | 211 | 212 | 210 | 165 | 165 |
| paternal half sibs |  | 209 | 210 | 208 | 163 | 163 |
| maternal grandmothers |  | 205 | 206 | 205 | 132 | 132 |
| maternal grandfathers |  | 118 | 119 | 118 | 78 | 78 |
| paternal grandmothers |  | 104 | 104 | 103 | 67 | 67 |
| paternal grandfathers |  | 76 | 76 | 75 | 49 | 49 |
| maximum pedigree depth |  | 7 | 7 | 7 | 6 | 6 |
| founders |  | 77 | 77 | 77 | 67 | 67 |
| mean maternal sibsip size |  | 1.641791045 | 1.643564356 | 1.633663366 | 1.614379085 | 1.614379085 |
| mean paternal sibsip size |  | 2.131868132 | 2.130434783 | 2.119565217 | 2.041666667 | 2.041666667 |
| non-zero F |  | 4 | 4 | 4 | 0 | 0 |
| F > 0.125 |  | 0 | 0 | 0 | 0 | 0 |
| mean pairwise relatedness |  | 0.008061482 | 0.007998292 | 0.008002688 | 0.008517589 | 0.008517589 |
| pairwise relatedness>=0.125 |  | 0.027468563 | 0.027250727 | 0.027251926 | 0.029514061 | 0.029514061 |
| pairwise relatedness>=0.25 |  | 0.014687693 | 0.014581173 | 0.014600712 | 0.016910133 | 0.016910133 |
| pairwise relatedness>=0.5 |  | 0.005421563 | 0.005389136 | 0.005407291 | 0.00655868 | 0.00655868 |
|  |  |  |  |  |  |  |
| **Pedigree 2** |  | **Foreleg** | **Hindleg** | **Weight** | **Metacarpal length** | **Jaw length** |
| records |  | 531 | 535 | 533 | 468 | 468 |
| maternities |  | 404 | 407 | 405 | 342 | 342 |
| paternities |  | 316 | 319 | 318 | 267 | 267 |
| full sibs |  | 4 | 4 | 4 | 3 | 3 |
| maternal sibs |  | 242 | 246 | 244 | 188 | 188 |
| maternal half sibs |  | 238 | 242 | 240 | 185 | 185 |
| paternal sibs |  | 381 | 384 | 381 | 284 | 284 |
| paternal half sibs |  | 377 | 380 | 377 | 281 | 281 |
| maternal grandmothers |  | 265 | 266 | 264 | 215 | 215 |
| maternal grandfathers |  | 220 | 222 | 221 | 175 | 175 |
| paternal grandmothers |  | 171 | 173 | 172 | 126 | 126 |
| paternal grandfathers |  | 147 | 147 | 146 | 107 | 107 |
| maximum pedigree depth |  | 8 | 8 | 8 | 8 | 8 |
| founders |  | 86 | 86 | 86 | 85 | 85 |
| mean maternal sibsip size |  | 1.719148936 | 1.724576271 | 1.716101695 | 1.644230769 | 1.644230769 |
| mean paternal sibsip size |  | 2.257142857 | 2.262411348 | 2.255319149 | 2.136 | 2.136 |
| non-zero F |  | 17 | 17 | 17 | 9 | 9 |
| F > 0.125 |  | 1 | 1 | 1 | 1 | 1 |
| mean pairwise relatedness |  | 0.009357922 | 0.00929119 | 0.009292344 | 0.00887713 | 0.00887713 |
| pairwise relatedness>=0.125 |  | 0.030487155 | 0.030305576 | 0.030307946 | 0.02973151 | 0.02973151 |
| pairwise relatedness>=0.25 |  | 0.01527911 | 0.01517729 | 0.015199819 | 0.015602409 | 0.015602409 |
| pairwise relatedness>=0.5 |  | 0.005159365 | 0.005124436 | 0.005141841 | 0.005618697 | 0.005618697 |

**Table S4:** Estimates of variance components ($\sigma_{i}^{2})$and their ratios to phenotypic variance ($\frac{\sigma_{i}^{2}}{\sigma_{p}^{2}}$) for model 1. Estimates are shown as means with standard errors within parentheses. Variance components differed between models, and shown are maternal effect (V_M_), additive genetic effect (V_A_), birth year effect (V_BY_), measurement year effect (V_CY_), permanent environment effect (V_PE_) and the residual variance (V_R_). Models for jaw length in yearlings did not converge using pedigree 1.

|  |  |  | **Pedigree 1** | | | **Pedigree 2** | | | **Genomic relatedness** | | |
| --- | --- | --- | --- | --- | --- | --- | --- | --- | --- | --- | --- |
| **Variance component** | **Age class** | **Trait** | $\boldsymbol{\sigma}_{\boldsymbol{i}}^{\boldsymbol{2}}$ | $\frac{\boldsymbol{\sigma}_{\boldsymbol{i}}^{\boldsymbol{2}}}{\boldsymbol{\sigma}_{\boldsymbol{p}}^{\boldsymbol{2}}}$ | **P** | $\boldsymbol{\sigma}_{\boldsymbol{i}}^{\boldsymbol{2}}$ | $\frac{\boldsymbol{\sigma}_{\boldsymbol{i}}^{\boldsymbol{2}}}{\boldsymbol{\sigma}_{\boldsymbol{p}}^{\boldsymbol{2}}}$ | **P.value** | $\boldsymbol{\sigma}_{\boldsymbol{i}}^{\boldsymbol{2}}$ | $\frac{\boldsymbol{\sigma}_{\boldsymbol{i}}^{\boldsymbol{2}}}{\boldsymbol{\sigma}_{\boldsymbol{p}}^{\boldsymbol{2}}}$ | **P.value** |
| V_M_ | Neonates | Birthweight | 0.064 (0.007) | 0.202 (0.023) | <0.001 | 0.06 (0.006) | 0.201 (0.021) | <0.001 | 0.056 (0.006) | 0.199 (0.021) | <0.001 |
| V_A_ |  |  | 0.034 (0.011) | 0.106 (0.034) | <0.001 | 0.027 (0.008) | 0.091 (0.026) | <0.001 | 0.016 (0.005) | 0.059 (0.017) | <0.001 |
| V_BY_ |  |  | 0.056 (0.017) | 0.177 (0.045) | NA | 0.05 (0.015) | 0.167 (0.041) | <0.001 | 0.04 (0.012) | 0.142 (0.037) | NA |
| V_R_ |  |  | 0.164 (0.009) | 0.515 (NA) | NA | 0.162 (0.007) | 0.54 (NA) | NA | 0.168 (0.006) | 0.601 (NA) | NA |
| V_M_ | Lambs | Foreleg | 4.175 (1.397) | 0.068 (0.023) | 0.001 | 3.795 (1.228) | 0.063 (0.021) | 0.001 | 3.685 (1.166) | 0.062 (0.02) | 0.001 |
| V_A_ |  |  | 6.634 (2.644) | 0.108 (0.043) | 0.003 | 9.321 (2.386) | 0.155 (0.041) | <0.001 | 8.634 (1.726) | 0.145 (0.031) | <0.001 |
| V_BY_ |  |  | 18.099 (5.925) | 0.293 (0.068) | NA | 17.105 (5.234) | 0.285 (0.063) | NA | 18.091 (5.599) | 0.303 (0.066) | NA |
| V_R_ |  |  | 32.793 (2.392) | 0.531 (NA) | NA | 29.75 (2.036) | 0.496 (NA) | NA | 29.266 (1.634) | 0.49 (NA) | NA |
| V_M_ |  | Hindleg | 7.375 (2.596) | 0.077 (0.027) | 0.002 | 6.556 (2.285) | 0.068 (0.024) | 0.002 | 6.278 (2.183) | 0.066 (0.023) | 0.002 |
| V_A_ |  |  | 13.542 (5.099) | 0.141 (0.052) | 0.002 | 18.817 (4.722) | 0.196 (0.048) | <0.001 | 14.755 (3.244) | 0.155 (0.033) | <0.001 |
| V_BY_ |  |  | 11.163 (3.894) | 0.116 (0.036) | NA | 12.055 (3.888) | 0.126 (0.036) | NA | 13.602 (4.483) | 0.143 (0.041) | NA |
| V_R_ |  |  | 63.78 (4.539) | 0.665 (NA) | NA | 58.568 (3.976) | 0.61 (NA) | NA | 60.783 (3.198) | 0.637 (NA) | NA |
| V_M_ |  | Weight | 0.687 (0.164) | 0.11 (0.027) | <0.001 | 0.639 (0.145) | 0.1 (0.023) | <0.001 | 0.581 (0.136) | 0.092 (0.022) | <0.001 |
| V_A_ |  |  | 0.64 (0.273) | 0.102 (0.044) | 0.007 | 0.744 (0.227) | 0.116 (0.036) | <0.001 | 0.655 (0.163) | 0.104 (0.026) | <0.001 |
| V_BY_ |  |  | 1.411 (0.44) | 0.225 (0.055) | NA | 1.583 (0.461) | 0.248 (0.055) | NA | 1.707 (0.517) | 0.271 (0.06) | NA |
| V_R_ |  |  | 3.529 (0.245) | 0.563 (NA) | NA | 3.418 (0.205) | 0.535 (NA) | NA | 3.361 (0.171) | 0.533 (NA) | NA |
| V_M_ |  | Metacarpal length | 2.657 (0.968) | 0.13 (0.047) | 0.005 | 1.637 (0.786) | 0.081 (0.039) | 0.045 | 2.999 (0.745) | 0.149 (0.036) | <0.001 |
| V_A_ |  |  | 7.336 (2.333) | 0.36 (0.11) | <0.001 | 10.305 (2.057) | 0.509 (0.092) | <0.001 | 5.874 (1.108) | 0.292 (0.051) | <0.001 |
| V_BY_ |  |  | 0.782 (0.433) | 0.038 (0.021) | NA | 0.944 (0.457) | 0.047 (0.022) | NA | 1.018 (0.493) | 0.051 (0.023) | NA |
| V_R_ |  |  | 9.612 (1.743) | 0.471 (NA) | NA | 7.353 (1.396) | 0.363 (NA) | NA | 10.217 (0.89) | 0.508 (NA) | NA |
| V_M_ |  | Jaw length | 2.917 (1.023) | 0.128 (0.044) | 0.004 | 3.22 (0.835) | 0.145 (0.037) | <0.001 | 3.698 (0.786) | 0.167 (0.034) | <0.001 |
| V_A_ |  |  | 8.288 (2.552) | 0.364 (0.108) | <0.001 | 6.73 (1.803) | 0.303 (0.078) | <0.001 | 5.105 (1.095) | 0.23 (0.047) | <0.001 |
| V_BY_ |  |  | 1.583 (0.731) | 0.07 (0.03) | NA | 2.028 (0.824) | 0.091 (0.034) | NA | 2.255 (0.967) | 0.102 (0.04) | NA |
| V_R_ |  |  | 9.967 (1.874) | 0.438 (NA) | NA | 10.219 (1.312) | 0.46 (NA) | NA | 11.145 (0.908) | 0.502 (NA) | NA |
| V_M_ | Yearlings | Foreleg | 3.651 (1.862) | 0.062 (0.033) | 0.046 | 5.422 (1.84) | 0.092 (0.033) | 0.002 | 5.426 (1.814) | 0.094 (0.033) | 0.002 |
| V_A_ |  |  | 7.38 (3.135) | 0.126 (0.055) | 0.006 | 9.222 (3.021) | 0.157 (0.054) | <0.001 | 6.24 (2.241) | 0.108 (0.04) | 0.001 |
| V_BY_ |  |  | 24.266 (8.137) | 0.414 (0.083) | NA | 23.513 (7.511) | 0.401 (0.078) | NA | 24.233 (7.741) | 0.418 (0.079) | NA |
| V_R_ |  |  | 23.341 (2.904) | 0.398 (NA) | NA | 20.49 (2.609) | 0.349 (NA) | NA | 22.084 (2.231) | 0.381 (NA) | NA |
| V_M_ |  | Hindleg | 8.869 (2.899) | 0.141 (0.045) | <0.001 | 11.08 (2.948) | 0.168 (0.043) | <0.001 | 12.267 (3.035) | 0.186 (0.044) | <0.001 |
| V_A_ |  |  | 16.672 (5.423) | 0.266 (0.083) | 0.001 | 20.323 (5.349) | 0.307 (0.075) | <0.001 | 16.027 (4.29) | 0.243 (0.061) | <0.001 |
| V_BY_ |  |  | 7.117 (2.866) | 0.113 (0.041) | NA | 6.969 (2.722) | 0.105 (0.037) | NA | 7.066 (2.797) | 0.107 (0.038) | NA |
| V_R_ |  |  | 30.122 (4.473) | 0.48 (NA) | NA | 27.761 (4.166) | 0.42 (NA) | NA | 30.611 (3.579) | 0.464 (NA) | NA |
| V_M_ |  | Weight | 0.8 (0.333) | 0.084 (0.036) | 0.007 | 0.941 (0.322) | 0.099 (0.034) | 0.001 | 0.993 (0.325) | 0.111 (0.037) | <0.001 |
| V_A_ |  |  | 2.033 (0.666) | 0.213 (0.069) | 0.001 | 1.808 (0.584) | 0.19 (0.061) | <0.001 | 1.343 (0.432) | 0.15 (0.048) | <0.001 |
| V_BY_ |  |  | 2.694 (0.91) | 0.283 (0.07) | NA | 2.617 (0.847) | 0.275 (0.065) | NA | 2.25 (0.748) | 0.251 (0.063) | NA |
| V_R_ |  |  | 4.005 (0.548) | 0.42 (NA) | NA | 4.159 (0.491) | 0.437 (NA) | NA | 4.389 (0.422) | 0.489 (NA) | NA |
| V_M_ |  | Metacarpal length | 2.23 (3.135) | 0.132 (0.184) | 0.464 | 0 (0) | 0 (0) | 1 | 0.478 (2.667) | 0.028 (0.155) | 0.859 |
| V_A_ |  |  | 4.003 (6.949) | 0.236 (0.407) | 0.553 | 10.7 (4.886) | 0.618 (0.26) | 0.009 | 9.777 (3.97) | 0.567 (0.207) | 0.003 |
| V_BY_ |  |  | 0.057 (0.383) | 0.003 (0.023) | NA | 0 (0) | 0 (0) | NA | 0 (0) | 0 (0) | NA |
| V_R_ |  |  | 10.651 (5.944) | 0.629 (NA) | NA | 6.606 (4.38) | 0.382 (NA) | NA | 6.991 (3.679) | 0.405 (NA) | NA |
| V_M_ |  | Jaw length | NA | NA | NA | 2.418 (3.34) | 0.089 (0.124) | 0.517 | 4.597 (2.771) | 0.191 (0.114) | 0.117 |
| V_A_ |  |  | NA | NA | NA | 18.167 (7.371) | 0.672 (0.262) | 0.002 | 14.968 (4.941) | 0.623 (0.184) | <0.001 |
| V_BY_ |  |  | NA | NA | NA | 5.177 (3.161) | 0.192 (0.098) | NA | 3.081 (2.131) | 0.128 (0.08) | NA |
| V_R_ |  |  | NA | NA | NA | 1.262 (5.097) | 0.047 (NA) | NA | 1.389 (3.618) | 0.058 (NA) | NA |
| V_M_ | Adults | Foreleg | 0 (0) | 0 (0) | 1 | 0.239 (0.723) | 0.005 (0.016) | 0.734 | 1.013 (0.773) | 0.023 (0.018) | 0.161 |
| V_A_ |  |  | 12.714 (2.014) | 0.291 (0.052) | <0.001 | 13.001 (1.861) | 0.296 (0.049) | <0.001 | 11.162 (1.667) | 0.257 (0.044) | <0.001 |
| V_PE_ |  |  | 5.717 (1.42) | 0.131 (0.036) | NA | 5.103 (1.356) | 0.116 (0.034) | NA | 5.78 (1.198) | 0.133 (0.032) | NA |
| V_CY_ |  |  | 16.196 (4.912) | 0.37 (0.072) | NA | 16.256 (4.923) | 0.37 (0.072) | NA | 16.155 (4.897) | 0.372 (0.072) | NA |
| V_BY_ |  |  | 0.77 (0.435) | 0.018 (0.01) | NA | 0.75 (0.4) | 0.017 (0.009) | NA | 0.766 (0.4) | 0.018 (0.009) | NA |
| V_R_ |  |  | 8.354 (0.314) | 0.191 (NA) | NA | 8.576 (0.31) | 0.195 (NA) | NA | 8.551 (0.313) | 0.197 (NA) | NA |
| V_M_ |  | Hindleg | 1.438 (1.544) | 0.031 (0.033) | 0.323 | 2.993 (1.543) | 0.063 (0.032) | 0.037 | 4.117 (1.539) | 0.087 (0.032) | 0.003 |
| V_A_ |  |  | 21.772 (3.611) | 0.468 (0.065) | <0.001 | 21.575 (3.356) | 0.458 (0.058) | <0.001 | 20.769 (3.008) | 0.441 (0.051) | <0.001 |
| V_PE_ |  |  | 13.974 (2.775) | 0.301 (0.064) | NA | 12.849 (2.472) | 0.273 (0.057) | NA | 12.184 (2.067) | 0.259 (0.048) | NA |
| V_CY_ |  |  | 0.81 (0.289) | 0.017 (0.006) | NA | 0.903 (0.319) | 0.019 (0.007) | NA | 0.831 (0.294) | 0.018 (0.006) | NA |
| V_BY_ |  |  | 2.126 (1.026) | 0.046 (0.021) | NA | 2.225 (1.011) | 0.047 (0.021) | NA | 2.418 (1.054) | 0.051 (0.021) | NA |
| V_R_ |  |  | 6.362 (0.232) | 0.137 (NA) | NA | 6.601 (0.232) | 0.14 (NA) | NA | 6.729 (0.24) | 0.143 (NA) | NA |
| V_M_ |  | Weight | 0.436 (0.29) | 0.049 (0.033) | 0.093 | 0.521 (0.283) | 0.057 (0.031) | 0.039 | 0.523 (0.276) | 0.057 (0.03) | 0.034 |
| V_A_ |  |  | 2.728 (0.591) | 0.31 (0.061) | <0.001 | 2.494 (0.535) | 0.273 (0.054) | <0.001 | 2.676 (0.497) | 0.294 (0.048) | <0.001 |
| V_PE_ |  |  | 2.784 (0.49) | 0.316 (0.058) | NA | 3.044 (0.451) | 0.333 (0.051) | NA | 2.883 (0.404) | 0.317 (0.047) | NA |
| V_CY_ |  |  | 0.604 (0.207) | 0.068 (0.022) | NA | 0.754 (0.252) | 0.082 (0.026) | NA | 0.684 (0.231) | 0.075 (0.024) | NA |
| V_BY_ |  |  | 0.142 (0.102) | 0.016 (0.012) | NA | 0.175 (0.11) | 0.019 (0.012) | NA | 0.2 (0.118) | 0.022 (0.013) | NA |
| V_R_ |  |  | 2.12 (0.077) | 0.241 (NA) | NA | 2.162 (0.075) | 0.236 (NA) | NA | 2.141 (0.076) | 0.235 (NA) | NA |
| V_M_ |  | Metacarpal length | 0 (0) | 0 (0) | 1 | 0.274 (0.661) | 0.018 (0.043) | 0.665 | 0.462 (0.682) | 0.03 (0.044) | 0.48 |
| V_A_ |  |  | 10.44 (1.763) | 0.668 (0.086) | <0.001 | 9.713 (1.59) | 0.631 (0.078) | <0.001 | 9.287 (1.491) | 0.594 (0.07) | <0.001 |
| V_BY_ |  |  | 0.177 (0.208) | 0.011 (0.013) | NA | 0.161 (0.186) | 0.01 (0.012) | NA | 0.174 (0.189) | 0.011 (0.012) | NA |
| V_R_ |  |  | 5.013 (1.241) | 0.321 (NA) | NA | 5.251 (1.166) | 0.341 (NA) | NA | 5.703 (1.029) | 0.365 (NA) | NA |
| V_M_ |  | Jaw length | 0 (0) | 0 (0) | 1 | 0.177 (0.733) | 0.01 (0.042) | 0.821 | 0.882 (0.832) | 0.05 (0.047) | 0.306 |
| V_A_ |  |  | 12.881 (2.011) | 0.729 (0.084) | <0.001 | 11.869 (1.832) | 0.677 (0.076) | <0.001 | 9.895 (1.696) | 0.556 (0.072) | <0.001 |
| V_BY_ |  |  | 0.331 (0.285) | 0.019 (0.016) | NA | 0.276 (0.251) | 0.016 (0.014) | NA | 0.191 (0.236) | 0.011 (0.013) | NA |
| V_R_ |  |  | 4.47 (1.344) | 0.253 (NA) | NA | 5.219 (1.338) | 0.298 (NA) | NA | 6.825 (1.236) | 0.384 (NA) | NA |

**Table S5:** Estimates of variance components ($\sigma_{i}^{2})$and their ratios to phenotypic variance ($\frac{\sigma_{i}^{2}}{\sigma_{p}^{2}}$) for model 2. Estimates are shown as means with standard errors within parentheses. Variance components differed between models, and shown are maternal genetic effect (V_MA_), maternal environment effect (V_ME_), additive genetic effect (V_A_), birth year effect (V_BY_), measurement year effect (V_CY_), permanent environment effect (V_PE_) and the residual variance (V_R_). Models for jaw length in yearlings did not converge using pedigree 1.

|  |  |  | **Pedigree 1** | | | **Pedigree 2** | | | **Genomic relatedness** | | |
| --- | --- | --- | --- | --- | --- | --- | --- | --- | --- | --- | --- |
| **Variance component** | **Age class** | **Trait** | $\boldsymbol{\sigma}_{\boldsymbol{i}}^{\boldsymbol{2}}$ | $\frac{\boldsymbol{\sigma}_{\boldsymbol{i}}^{\boldsymbol{2}}}{\boldsymbol{\sigma}_{\boldsymbol{p}}^{\boldsymbol{2}}}$ | **P** | $\boldsymbol{\sigma}_{\boldsymbol{i}}^{\boldsymbol{2}}$ | $\frac{\boldsymbol{\sigma}_{\boldsymbol{i}}^{\boldsymbol{2}}}{\boldsymbol{\sigma}_{\boldsymbol{p}}^{\boldsymbol{2}}}$ | **P.value** | $\boldsymbol{\sigma}_{\boldsymbol{i}}^{\boldsymbol{2}}$ | $\frac{\boldsymbol{\sigma}_{\boldsymbol{i}}^{\boldsymbol{2}}}{\boldsymbol{\sigma}_{\boldsymbol{p}}^{\boldsymbol{2}}}$ | **P.value** |
| V_MA_ | Neonates | Birthweight | 0.014 (0.01) | 0.046 (0.033) | 0.134 | 0.023 (0.01) | 0.077 (0.031) | 0.009 | 0.045 (0.01) | 0.155 (0.033) | <0.001 |
| V_ME_ |  |  | 0.05 (0.01) | 0.16 (0.033) | <0.001 | 0.042 (0.009) | 0.14 (0.029) | <0.001 | 0.024 (0.008) | 0.085 (0.027) | 0.001 |
| V_A_ |  |  | 0.03 (0.011) | 0.098 (0.034) | <0.001 | 0.024 (0.008) | 0.079 (0.025) | <0.001 | 0.012 (0.005) | 0.041 (0.016) | <0.001 |
| V_BY_ |  |  | 0.053 (0.016) | 0.17 (0.043) | NA | 0.05 (0.014) | 0.164 (0.04) | NA | 0.037 (0.011) | 0.127 (0.034) | NA |
| V_R_ |  |  | 0.163 (0.009) | 0.526 (NA) | NA | 0.163 (0.007) | 0.539 (NA) | NA | 0.17 (0.006) | 0.591 (NA) | NA |
| V_MA_ | Lambs | Foreleg | 1.844 (2.062) | 0.03 (0.033) | 0.374 | 1.975 (1.834) | 0.033 (0.031) | 0.327 | 3.524 (1.695) | 0.059 (0.028) | 0.025 |
| V_ME_ |  |  | 2.795 (1.966) | 0.045 (0.032) | 0.164 | 2.368 (1.679) | 0.039 (0.028) | 0.191 | 1.581 (1.485) | 0.026 (0.025) | 0.3 |
| V_A_ |  |  | 5.584 (2.828) | 0.09 (0.046) | 0.022 | 8.424 (2.5) | 0.14 (0.042) | <0.001 | 7.749 (1.792) | 0.13 (0.031) | <0.001 |
| V_BY_ |  |  | 18.029 (5.903) | 0.292 (0.068) | NA | 17.063 (5.223) | 0.284 (0.063) | NA | 17.47 (5.435) | 0.292 (0.065) | NA |
| V_R_ |  |  | 33.452 (2.499) | 0.542 (NA) | NA | 30.234 (2.093) | 0.503 (NA) | NA | 29.47 (1.688) | 0.493 (NA) | NA |
| V_MA_ |  | Hindleg | 6.827 (4.229) | 0.071 (0.044) | 0.113 | 6.59 (3.63) | 0.068 (0.037) | 0.074 | 9.763 (3.411) | 0.101 (0.034) | 0.001 |
| V_ME_ |  |  | 2.88 (3.591) | 0.03 (0.037) | 0.433 | 2.11 (3.013) | 0.022 (0.031) | 0.504 | 0.093 (2.684) | 0.001 (0.028) | 0.974 |
| V_A_ |  |  | 8.638 (5.32) | 0.09 (0.055) | 0.076 | 15.493 (4.83) | 0.161 (0.049) | <0.001 | 12.077 (3.298) | 0.124 (0.033) | <0.001 |
| V_BY_ |  |  | 11.03 (3.852) | 0.115 (0.036) | NA | 12.007 (3.876) | 0.125 (0.035) | NA | 13.282 (4.41) | 0.137 (0.04) | NA |
| V_R_ |  |  | 66.647 (4.714) | 0.694 (NA) | NA | 60.205 (4.058) | 0.624 (NA) | NA | 61.824 (3.305) | 0.637 (NA) | NA |
| V_MA_ |  | Weight | 0.659 (0.261) | 0.104 (0.041) | 0.006 | 0.611 (0.217) | 0.095 (0.034) | 0.001 | 0.649 (0.203) | 0.103 (0.032) | <0.001 |
| V_ME_ |  |  | 0.219 (0.221) | 0.035 (0.035) | 0.332 | 0.206 (0.184) | 0.032 (0.029) | 0.27 | 0.171 (0.167) | 0.027 (0.027) | 0.315 |
| V_A_ |  |  | 0.234 (0.263) | 0.037 (0.042) | 0.361 | 0.421 (0.215) | 0.066 (0.034) | 0.016 | 0.495 (0.166) | 0.078 (0.027) | <0.001 |
| V_BY_ |  |  | 1.419 (0.443) | 0.225 (0.055) | NA | 1.585 (0.462) | 0.247 (0.055) | NA | 1.595 (0.488) | 0.253 (0.058) | NA |
| V_R_ |  |  | 3.778 (0.248) | 0.599 (NA) | NA | 3.596 (0.206) | 0.56 (NA) | NA | 3.404 (0.178) | 0.539 (NA) | NA |
| V_MA_ |  | Metacarpal length | 3.685 (1.01) | 0.18 (0.047) | 0.003 | 2.835 (0.855) | 0.139 (0.041) | 0.001 | 3.512 (0.807) | 0.172 (0.037) | <0.001 |
| V_ME_ |  |  | 0 (0) | 0 (0) | 1 | 0 (0) | 0 (0) | 1 | 0 (0) | 0 (0) | 1 |
| V_A_ |  |  | 4.85 (2.193) | 0.236 (0.105) | 0.002 | 8.175 (1.937) | 0.402 (0.09) | <0.001 | 5.472 (1.115) | 0.268 (0.051) | <0.001 |
| V_BY_ |  |  | 0.816 (0.443) | 0.04 (0.021) | NA | 0.958 (0.458) | 0.047 (0.022) | NA | 1.084 (0.52) | 0.053 (0.024) | NA |
| V_R_ |  |  | 11.173 (1.699) | 0.544 (NA) | NA | 8.391 (1.357) | 0.412 (NA) | NA | 10.352 (0.87) | 0.507 (NA) | NA |
| V_MA_ |  | Jaw length | 3.162 (1.585) | 0.139 (0.068) | 0.041 | 3.142 (1.224) | 0.141 (0.054) | 0.004 | 2.637 (1.03) | 0.119 (0.045) | 0.003 |
| V_ME_ |  |  | 0.833 (1.334) | 0.037 (0.059) | 0.553 | 1.064 (1.045) | 0.048 (0.047) | 0.321 | 1.443 (0.947) | 0.065 (0.043) | 0.119 |
| V_A_ |  |  | 5.55 (2.555) | 0.243 (0.111) | 0.01 | 4.527 (1.693) | 0.203 (0.075) | 0.001 | 4.622 (1.11) | 0.208 (0.048) | <0.001 |
| V_BY_ |  |  | 1.533 (0.708) | 0.067 (0.029) | NA | 1.976 (0.801) | 0.089 (0.033) | NA | 2.114 (0.915) | 0.095 (0.038) | NA |
| V_R_ |  |  | 11.738 (1.925) | 0.514 (NA) | NA | 11.546 (1.296) | 0.519 (NA) | NA | 11.386 (0.937) | 0.513 (NA) | NA |
| V_MA_ | Yearlings | Foreleg | 2.746 (2.805) | 0.047 (0.048) | 0.327 | 4.582 (2.735) | 0.078 (0.047) | 0.074 | 4.355 (2.309) | 0.077 (0.042) | 0.028 |
| V_ME_ |  |  | 1.508 (2.644) | 0.026 (0.045) | 0.586 | 1.724 (2.475) | 0.029 (0.042) | 0.505 | 2.386 (2.251) | 0.042 (0.04) | 0.294 |
| V_A_ |  |  | 5.161 (3.443) | 0.088 (0.06) | 0.106 | 6.331 (3.13) | 0.108 (0.054) | 0.031 | 4.106 (2.246) | 0.073 (0.041) | 0.039 |
| V_BY_ |  |  | 24.132 (8.091) | 0.414 (0.083) | NA | 23.48 (7.496) | 0.402 (0.078) | NA | 22.889 (7.339) | 0.407 (0.079) | NA |
| V_R_ |  |  | 24.81 (3.113) | 0.425 (NA) | NA | 22.353 (2.748) | 0.382 (NA) | NA | 22.473 (2.286) | 0.4 (NA) | NA |
| V_MA_ |  | Hindleg | 5.356 (4.785) | 0.086 (0.076) | 0.26 | 6.226 (4.483) | 0.094 (0.067) | 0.132 | 6.697 (4.011) | 0.104 (0.061) | 0.057 |
| V_ME_ |  |  | 5.008 (4.177) | 0.08 (0.067) | 0.215 | 6.174 (3.999) | 0.093 (0.06) | 0.106 | 7.913 (3.753) | 0.123 (0.058) | 0.019 |
| V_A_ |  |  | 12.798 (5.958) | 0.205 (0.093) | 0.037 | 17.976 (5.578) | 0.271 (0.08) | 0.001 | 13.939 (4.291) | 0.217 (0.064) | <0.001 |
| V_BY_ |  |  | 6.988 (2.825) | 0.112 (0.041) | NA | 6.993 (2.728) | 0.106 (0.037) | NA | 6.651 (2.656) | 0.103 (0.038) | NA |
| V_R_ |  |  | 32.328 (4.82) | 0.517 (NA) | NA | 28.891 (4.316) | 0.436 (NA) | NA | 29.125 (3.529) | 0.453 (NA) | NA |
| V_MA_ |  | Weight | 0.613 (0.575) | 0.064 (0.06) | 0.277 | 0.593 (0.495) | 0.062 (0.052) | 0.204 | 0.644 (0.421) | 0.073 (0.048) | 0.084 |
| V_ME_ |  |  | 0.365 (0.494) | 0.038 (0.052) | 0.457 | 0.523 (0.447) | 0.055 (0.047) | 0.224 | 0.541 (0.399) | 0.061 (0.045) | 0.133 |
| V_A_ |  |  | 1.74 (0.724) | 0.182 (0.075) | 0.012 | 1.489 (0.621) | 0.156 (0.065) | 0.008 | 1.165 (0.451) | 0.132 (0.051) | 0.001 |
| V_BY_ |  |  | 2.7 (0.913) | 0.282 (0.07) | NA | 2.611 (0.845) | 0.274 (0.065) | NA | 2.115 (0.715) | 0.24 (0.063) | NA |
| V_R_ |  |  | 4.155 (0.58) | 0.434 (NA) | NA | 4.327 (0.514) | 0.453 (NA) | NA | 4.336 (0.43) | 0.493 (NA) | NA |
| V_MA_ |  | Metacarpal length | 0 (0) | 0 (0) | 1 | 0 (0) | 0 (0) | 1 | 0 (0) | 0 (0) | 1 |
| V_ME_ |  |  | 2.23 (3.135) | 0.132 (0.184) | 0.464 | 0 (0) | 0 (0) | 1 | 0.425 (2.745) | 0.024 (0.157) | 0.877 |
| V_A_ |  |  | 4.002 (6.951) | 0.236 (0.407) | 0.553 | 10.7 (4.886) | 0.618 (0.26) | 0.009 | 10.237 (4.105) | 0.586 (0.211) | 0.003 |
| V_BY_ |  |  | 0.057 (0.383) | 0.003 (0.023) | NA | 0 (0) | 0 (0) | NA | 0 (0) | 0 (0) | NA |
| V_R_ |  |  | 10.649 (5.944) | 0.629 (NA) | NA | 6.606 (4.38) | 0.382 (NA) | NA | 6.81 (3.793) | 0.39 (NA) | NA |
| V_MA_ |  | Jaw length | NA | NA | NA | 0 (0) | 0 (0) | 1 | 0 (0) | 0 (0) | 1 |
| V_ME_ |  |  | NA | NA | NA | 2.417 (3.338) | 0.09 (0.124) | 0.526 | 4.967 (2.694) | 0.208 (0.112) | 0.109 |
| V_A_ |  |  | NA | NA | NA | 18.154 (7.42) | 0.672 (0.263) | 0.002 | 14.566 (5.124) | 0.61 (0.19) | 0.001 |
| V_BY_ |  |  | NA | NA | NA | 5.17 (3.156) | 0.191 (0.098) | NA | 3.798 (2.498) | 0.159 (0.091) | NA |
| V_R_ |  |  | NA | NA | NA | 1.26 (5.098) | 0.047 (NA) | NA | 0.547 (3.599) | 0.023 (NA) | NA |
| V_MA_ | Adults | Foreleg | 0.348 (0.987) | 0.008 (0.023) | 0.71 | 0.91 (0.906) | 0.021 (0.021) | 0.289 | 1.737 (1.268) | 0.04 (0.029) | 0.143 |
| V_ME_ |  |  | 0 (0) | 0 (0) | 1 | 0 (0) | 0 (0) | 1 | 0.228 (1.022) | 0.005 (0.023) | 0.824 |
| V_A_ |  |  | 12.501 (2.127) | 0.286 (0.054) | <0.001 | 12.584 (1.936) | 0.286 (0.05) | <0.001 | 10.922 (1.787) | 0.25 (0.046) | <0.001 |
| V_PE_ |  |  | 5.62 (1.451) | 0.128 (0.037) | <0.001 | 4.966 (1.259) | 0.113 (0.032) | <0.001 | 5.412 (1.24) | 0.124 (0.032) | <0.001 |
| V_BY_ |  |  | 0.759 (0.43) | 0.017 (0.01) | NA | 0.733 (0.392) | 0.017 (0.009) | NA | 0.814 (0.416) | 0.019 (0.01) | NA |
| V_CY_ |  |  | 16.164 (4.902) | 0.369 (0.072) | NA | 16.218 (4.912) | 0.369 (0.071) | NA | 16.079 (4.882) | 0.369 (0.072) | NA |
| V_R_ |  |  | 8.355 (0.314) | 0.191 (NA) | NA | 8.576 (0.31) | 0.195 (NA) | NA | 8.434 (0.314) | 0.193 (NA) | NA |
| V_MA_ |  | Hindleg | 3.258 (2.041) | 0.07 (0.043) | 0.163 | 3.665 (2.863) | 0.077 (0.059) | 0.19 | 1.688 (2.381) | 0.036 (0.05) | 0.511 |
| V_ME_ |  |  | 0 (0) | 0 (0) | 1 | 0.724 (2.167) | 0.015 (0.046) | 0.742 | 3.37 (2.146) | 0.071 (0.045) | 0.123 |
| V_A_ |  |  | 20.122 (3.842) | 0.431 (0.072) | <0.001 | 20.237 (3.583) | 0.426 (0.066) | <0.001 | 20.894 (3.196) | 0.441 (0.055) | <0.001 |
| V_PE_ |  |  | 14.169 (2.672) | 0.303 (0.062) | <0.001 | 13.272 (2.564) | 0.279 (0.058) | <0.001 | 11.671 (2.133) | 0.246 (0.049) | <0.001 |
| V_BY_ |  |  | 2.009 (0.985) | 0.043 (0.02) | NA | 2.124 (0.977) | 0.045 (0.02) | NA | 2.163 (0.976) | 0.046 (0.02) | NA |
| V_CY_ |  |  | 0.81 (0.289) | 0.017 (0.006) | NA | 0.902 (0.319) | 0.019 (0.007) | NA | 0.832 (0.295) | 0.018 (0.006) | NA |
| V_R_ |  |  | 6.362 (0.232) | 0.136 (NA) | NA | 6.6 (0.232) | 0.139 (NA) | NA | 6.802 (0.246) | 0.143 (NA) | NA |
| V_MA_ |  | Weight | 0.4 (0.524) | 0.045 (0.059) | 0.435 | 0.759 (0.333) | 0.082 (0.035) | 0.09 | 0.707 (0.435) | 0.076 (0.046) | 0.081 |
| V_ME_ |  |  | 0.163 (0.431) | 0.018 (0.049) | 0.702 | 0 (0) | 0 (0) | 1 | 0.084 (0.367) | 0.009 (0.04) | 0.82 |
| V_A_ |  |  | 2.591 (0.64) | 0.293 (0.068) | <0.001 | 2.27 (0.561) | 0.246 (0.057) | <0.001 | 2.584 (0.536) | 0.278 (0.052) | <0.001 |
| V_PE_ |  |  | 2.835 (0.508) | 0.32 (0.059) | <0.001 | 3.115 (0.439) | 0.338 (0.05) | <0.001 | 2.9 (0.427) | 0.312 (0.048) | <0.001 |
| V_BY_ |  |  | 0.141 (0.102) | 0.016 (0.011) | NA | 0.171 (0.108) | 0.018 (0.012) | NA | 0.203 (0.121) | 0.022 (0.013) | NA |
| V_CY_ |  |  | 0.603 (0.207) | 0.068 (0.022) | NA | 0.752 (0.252) | 0.082 (0.025) | NA | 0.667 (0.228) | 0.072 (0.023) | NA |
| V_R_ |  |  | 2.12 (0.077) | 0.239 (NA) | NA | 2.162 (0.075) | 0.234 (NA) | NA | 2.146 (0.077) | 0.231 (NA) | NA |
| V_MA_ |  | Metacarpal length | 0.007 (0.904) | 0 (0.058) | 0.993 | 0.496 (0.786) | 0.032 (0.051) | 0.604 | 0.782 (0.783) | 0.049 (0.049) | 0.418 |
| V_ME_ |  |  | 0 (0) | 0 (0) | 1 | 0 (0) | 0 (0) | 1 | 0 (0) | 0 (0) | 1 |
| V_A_ |  |  | 10.247 (2.027) | 0.657 (0.106) | <0.001 | 9.407 (1.673) | 0.61 (0.087) | <0.001 | 8.998 (1.583) | 0.568 (0.077) | <0.001 |
| V_BY_ |  |  | 0.202 (0.236) | 0.013 (0.015) | NA | 0.165 (0.187) | 0.011 (0.012) | NA | 0.204 (0.205) | 0.013 (0.013) | NA |
| V_R_ |  |  | 5.135 (1.361) | 0.329 (NA) | NA | 5.35 (1.086) | 0.347 (NA) | NA | 5.87 (1.007) | 0.37 (NA) | NA |
| V_MA_ |  | Jaw length | 0 (0) | 0 (0) | 1 | 0 (0) | 0 (0) | 1 | 0 (0) | 0 (0) | 1 |
| V_ME_ |  |  | 0 (0) | 0 (0) | 1 | 0.177 (0.733) | 0.01 (0.042) | 0.821 | 1.229 (0.88) | 0.068 (0.048) | 0.175 |
| V_A_ |  |  | 12.455 (2.079) | 0.73 (0.09) | <0.001 | 11.868 (1.832) | 0.677 (0.076) | <0.001 | 10.36 (1.784) | 0.573 (0.074) | <0.001 |
| V_BY_ |  |  | 0.492 (0.34) | 0.029 (0.02) | NA | 0.276 (0.251) | 0.016 (0.014) | NA | 0.246 (0.26) | 0.014 (0.014) | NA |
| V_R_ |  |  | 4.125 (1.406) | 0.242 (NA) | NA | 5.219 (1.338) | 0.298 (NA) | NA | 6.24 (1.275) | 0.345 (NA) | NA |

**Table S6:** Estimates of genetic and environmental covariance between adult body size traits. Estimates are shown as means with standard errors within parentheses.

| **Random effect** | **Traits** | **Pedigree 1** | | **Pedigree 2** | | **Genomic relatedness** | |
| --- | --- | --- | --- | --- | --- | --- | --- |
|  |  | **Covariance** | **Correlation** | **Covariance** | **Correlation** | **Covariance** | **Correlation** |
| Maternal | Foreleg-Weight | 0.089 (0.373) | 0.694 (4.842) | 0.51 (0.383) | 0.796 (0.359) | 0.912 (0.398) | 0.97 (0.216) |
|  | Hindleg-Weight | 1.05 (0.599) | 0.894 (0.241) | 1.566 (0.594) | 0.916 (0.156) | 1.86 (0.592) | 0.936 (0.139) |
|  | Hindleg-Foreleg | 0.132 (1.014) | 0.981 (12.353) | 1.651 (1.057) | 0.964 (0.128) | 2.938 (1.093) | 0.941 (0.059) |
|  | Hindleg-Metacarpal length | 1.258 (0.929) | 0.854 (0.288) | 1.382 (0.843) | 0.832 (0.256) | 1.494 (0.807) | 0.827 (0.232) |
|  | Foreleg-Metacarpal length | 0.095 (0.591) | 0.553 (2.201) | 0.422 (0.539) | 0.807 (0.484) | 0.61 (0.542) | 0.782 (0.338) |
|  | Weight-Metacarpal length | 0.298 (0.367) | 0.93 (1.765) | 0.079 (0.321) | 0.157 (0.592) | 0.234 (0.326) | 0.378 (0.457) |
|  | Hindleg-Jaw length | 0.519 (0.871) | 0.703 (0.778) | 1.348 (0.846) | 0.905 (0.382) | 1.933 (0.893) | 0.907 (0.283) |
|  | Foreleg-Jaw length | -0.201 (0.616) | -0.651 (3.104) | 0.366 (0.577) | 0.644 (0.747) | 0.779 (0.623) | 0.636 (0.365) |
|  | Weight-Jaw length | 0.141 (0.36) | 0.436 (0.979) | 0.457 (0.348) | 0.732 (0.477) | 0.633 (0.362) | 0.857 (0.383) |
|  | Metacarpal length-Jaw length | -0.009 (0.601) | -0.025 (1.684) | -0.044 (0.524) | -0.114 (1.466) | 0.3 (0.573) | 0.361 (0.559) |
| Additive genetic | Foreleg-Weight | 2.952 (0.868) | 0.504 (0.101) | 2.939 (0.797) | 0.535 (0.095) | 2.347 (0.713) | 0.446 (0.097) |
|  | Hindleg-Weight | 3.364 (1.194) | 0.448 (0.108) | 3.126 (1.077) | 0.453 (0.106) | 3.158 (0.968) | 0.455 (0.095) |
|  | Hindleg-Foreleg | 14.986 (2.521) | 0.927 (0.023) | 14.669 (2.338) | 0.925 (0.022) | 13.65 (2.1) | 0.944 (0.019) |
|  | Hindleg-Metacarpal length | 11.832 (2.095) | 0.833 (0.054) | 11.616 (1.821) | 0.873 (0.048) | 11.669 (1.671) | 0.889 (0.041) |
|  | Foreleg-Metacarpal length | 8.868 (1.469) | 0.857 (0.053) | 8.765 (1.296) | 0.893 (0.042) | 8.362 (1.193) | 0.91 (0.038) |
|  | Weight-Metacarpal length | 1.57 (0.794) | 0.302 (0.138) | 1.627 (0.678) | 0.34 (0.126) | 1.436 (0.63) | 0.292 (0.117) |
|  | Hindleg-Jaw length | 9.853 (2.143) | 0.642 (0.079) | 8.602 (1.925) | 0.548 (0.08) | 6.917 (1.716) | 0.492 (0.085) |
|  | Foreleg-Jaw length | 6.291 (1.535) | 0.569 (0.092) | 5.485 (1.371) | 0.473 (0.088) | 4.427 (1.229) | 0.442 (0.094) |
|  | Weight-Jaw length | 3.803 (0.869) | 0.657 (0.103) | 3.204 (0.767) | 0.59 (0.101) | 2.862 (0.706) | 0.545 (0.097) |
|  | Metacarpal length-Jaw length | 4.886 (1.533) | 0.436 (0.105) | 4.34 (1.279) | 0.41 (0.099) | 3.883 (1.182) | 0.414 (0.102) |
| Permanent environment | Foreleg-Weight | 3.179 (0.701) | 0.702 (0.095) | 3.023 (0.63) | 0.685 (0.088) | 3.046 (0.559) | 0.674 (0.077) |
|  | Hindleg-Weight | 4.846 (0.958) | 0.731 (0.078) | 4.811 (0.861) | 0.708 (0.071) | 4.38 (0.752) | 0.669 (0.066) |
|  | Hindleg-Foreleg | 10.717 (1.954) | 0.976 (0.03) | 9.608 (1.72) | 0.997 (0.03) | 9.162 (1.47) | 0.961 (0.028) |
|  | Hindleg-Metacarpal length | 6.516 (1.579) | 0.71 (0.081) | 6.29 (1.333) | 0.667 (0.071) | 6.169 (1.153) | 0.655 (0.065) |
|  | Foreleg-Metacarpal length | 5.02 (1.143) | 0.798 (0.082) | 4.702 (0.959) | 0.772 (0.071) | 4.908 (0.848) | 0.763 (0.063) |
|  | Weight-Metacarpal length | 1.688 (0.658) | 0.435 (0.15) | 1.931 (0.563) | 0.467 (0.119) | 1.948 (0.508) | 0.469 (0.107) |
|  | Hindleg-Jaw length | 5.882 (1.644) | 0.628 (0.107) | 5.523 (1.416) | 0.629 (0.105) | 6.398 (1.264) | 0.641 (0.083) |
|  | Foreleg-Jaw length | 4.376 (1.218) | 0.696 (0.129) | 3.818 (1.037) | 0.657 (0.128) | 4.462 (0.941) | 0.661 (0.101) |
|  | Weight-Jaw length | 1.415 (0.69) | 0.379 (0.15) | 1.59 (0.593) | 0.413 (0.125) | 1.939 (0.541) | 0.433 (0.099) |
| Year of measurement | Foreleg-Weight | 0.845 (0.761) | 0.257 (0.208) | 1.021 (0.849) | 0.277 (0.204) | 1.055 (0.835) | 0.294 (0.203) |
|  | Hindleg-Weight | 0.347 (0.215) | 0.438 (0.199) | 0.375 (0.243) | 0.41 (0.203) | 0.315 (0.224) | 0.372 (0.212) |
|  | Hindleg-Foreleg | 2.906 (1.041) | 0.773 (0.097) | 3.043 (1.11) | 0.749 (0.103) | 2.993 (1.089) | 0.754 (0.102) |
| Residual | Foreleg-Weight | 0.098 (0.111) | 0.023 (0.026) | 0.153 (0.11) | 0.036 (0.026) | 0.153 (0.111) | 0.036 (0.026) |
|  | Hindleg-Weight | 0.258 (0.095) | 0.07 (0.026) | 0.264 (0.094) | 0.07 (0.025) | 0.261 (0.096) | 0.069 (0.025) |
|  | Hindleg-Foreleg | 0.826 (0.195) | 0.115 (0.026) | 0.81 (0.194) | 0.108 (0.025) | 0.808 (0.198) | 0.107 (0.026) |
|  | Metacarpal length-Jaw length | 1.815 (1.15) | 0.39 (0.194) | 1.751 (0.929) | 0.326 (0.145) | 1.975 (0.841) | 0.311 (0.112) |

**Figure S1:** Correlations between pairwise relatedness estimates obtained using pedigree 1, pedigree 2 and genomic relatedness. The grey dashed lines indicate the y=x line, while the black solid lines indicate the model fit from a linear regression.


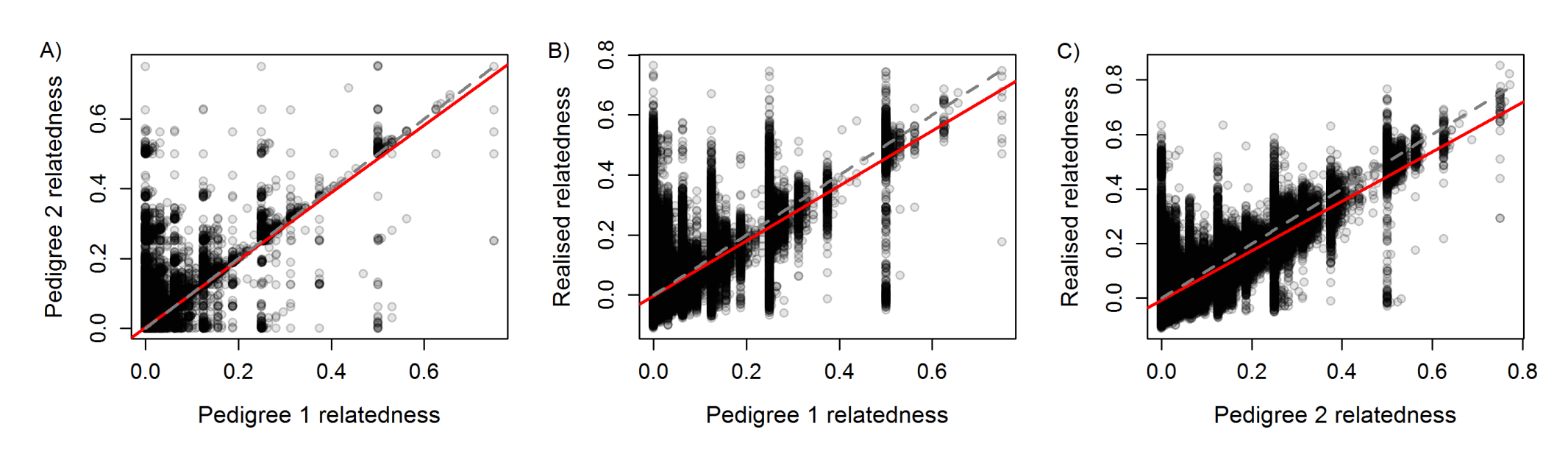

Supplement: Supplementary file 1 [file mec0023-3434-SD1.docx]
